# Behavior of Silicon Carbide Materials under Dry to Hydrothermal Conditions

**DOI:** 10.3390/nano11051351

**Published:** 2021-05-20

**Authors:** Nicolas Biscay, Lucile Henry, Tadafumi Adschiri, Masahiro Yoshimura, Cyril Aymonier

**Affiliations:** 1 CNRS, University of Bordeaux, Bordeaux INP, ICMCB, UMR 5026, 33600 Pessac, France; nicolas.biscay@icmcb.cnrs.fr (N.B.); lucile.henry@icmcb.cnrs.fr (L.H.); 2 WPI-Advanced Institute for Materials Research (WPI-AIMR), Tohoku University, 2-1-1 Katahira, Aoba-ku, Sendai 980-8577, Japan; tadafumi.ajiri.b1@tohoku.ac.jp; 3 Department of Materials Science and Engineering, National Cheng Kung University, No. 1, University Road, Tainan 70101, Taiwan; masahiroyoshimura75@gmail.com

**Keywords:** silicon carbide, wet oxidation, supercritical fluids, supercritical water oxidation, hydrothermal corrosion, nanocarbon films

## Abstract

Silicon carbide materials are excellent candidates for high-performance applications due to their outstanding thermomechanical properties and their strong corrosion resistance. SiC materials can be processed in various forms, from nanomaterials to continuous fibers. Common applications of SiC materials include the aerospace and nuclear fields, where the material is used in severely oxidative environments. Therefore, it is important to understand the kinetics of SiC oxidation and the parameters influencing them. The first part of this review focuses on the oxidation of SiC in dry air according to the Deal and Grove model showing that the oxidation behavior of SiC depends on the temperature and the time of oxidation. The oxidation rate can also be accelerated with the presence of H_2_O in the system due to its diffusion through the oxide scales. Therefore, wet oxidation is studied in the second part. The third part details the effect of hydrothermal media on the SiC materials that has been explained by different models, namely Yoshimura (1986), Hirayama (1989) and Allongue (1992). The last part of this review focuses on the hydrothermal corrosion of SiC materials from an application point of view and determine whether it is beneficial (manufacturing of materials) or detrimental (use of SiC in latest nuclear reactors).

## 1. Introduction

Ceramics have been used as dielectric, magnetic and optical materials. The oxide ceramics are chemically stable at high temperature and have good refractory properties but poor thermal-shock resistance. This is not the case for the non-oxide ceramics. Non-oxide ceramics show a high thermal conductivity, which leads to excellent thermal-shock resistance. Non-oxide ceramics are composed essentially of borides, nitrides and carbides, of which silicon carbide (SiC) is the most widely used. SiC was originally discovered in 1891 by Acheson under the name of “carborundum” [1]. SiC materials have low density, and they exhibit a high degree of hardness and toughness due to an important degree of crosslinking of covalent bond. These properties justify their use for not only aerospace and automotive parts, but also in nuclear applications. In 1975, Yajima et al. elaborated a process for producing SiC materials in a fiber shape by pyrolysis of organosilicon polymers [2]. This process allows for continuous fiber production, generating fibers with a small diameter and with good flexibility to be used for designing composite materials. Carbon-based materials reinforced with SiC fibers have higher mechanical properties: these then constitute thermostructural composites for high-performance applications [3].

At high temperatures, silicon carbide undergoes passive and active oxidation, which contribute to its degradation.

Passive oxidation is responsible for both the formation of a silica layer on the top of the surface and for the active oxidation for the release of volatile oxides. The material is not able to withstand high mechanical properties as the oxidation is occurs, and dramatic failures can result when exposed to stress. Moreover, in the aerospace field, water and corrosive gases are released by the propulsion system. This, along with high temperatures, is expected to enhance the degradation of SiC materials. However, SiC materials need to operate properly for a defined range of temperatures and various gas compositions. In that way, the whole oxidation process needs to be characterized, and all the influential parameters need to be well understood.

What kind of oxidation behavior will the material exhibit when exposed to dry atmosphere? Which parameters can influence the oxidation kinetics? For example, what is the influence of water on the oxidation behavior? What are the effects of hot water and pressurized atmospheres? How do high temperature and high pressure water modify the surface properties?

The aim of this paper is to review, firstly, the dry oxidation of SiC materials, as the literature has already provided a comprehensive background of this phenomena along with accurate kinetic models.

In the first part, the oxidation of SiC under dry conditions, and the Deal and Grove model for the passive oxidation of silicon, are explained. The parameters which can influence the oxidation behavior of SiC are studied. The nature of silica scale and oxidant species are discussed, as well as the influence of crystal faces and impurities, to lead to the conclusion of the rate-determining step of the SiC oxidation.

In the second part, the effect of water vapor onto SiC is studied and a mixed oxidation regime which is in competition with the passive oxidation regime, is expressed. Then, the parameters which influence the oxidation of SiC are reviewed.

The third part focuses on the importance of understanding the ability of water to accelerate SiC degradation. Hydrothermal conditions are disastrous for SiC materials and lead to chemical corrosion through three possible reactions: wet (air) oxidation, supercritical water oxidation and hydrolysis. Then, three models for the interaction of SiC with water are proposed, and their validity is assessed by the microstructural study of the corroded surface. Finally, another SiC corrosion mechanism is discussed which occurs under hydrothermal conditions—tribochemical corrosion.

The last part deals with supercritical water medium and its interaction with SiC materials. This interaction can be either profitable or detrimental depending on the desired application.

## 2. Dry Oxidation of Silicon Carbide Materials

**Figure 1:**
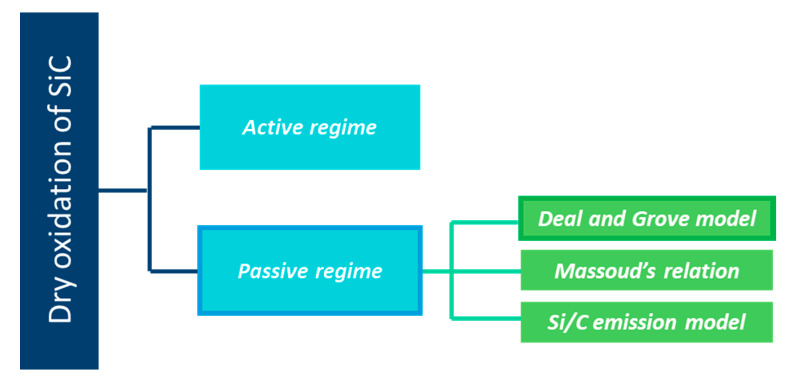
Layout of the dry oxidation of SiC part.

In Figure 1, a scheme is provided, detailing the layout of the following part.

As a silicon-based ceramic, silicon carbide is unstable in air. At high temperatures and under a dry atmosphere, SiC materials undergo passive (1) or active oxidation (2).

SiC (s) + 3/2 O_2_ (g) → SiO_2_ (s) + CO (g) (1)

SiC (s) + O_2_ (s) → SiO (g) + CO (g) (2)

The SiO_2_ layer formed according to Equation (1) at the surface has a low permeability to oxygen, so it can act as a protective barrier to prevent further oxidation of the bulk material. This protective effect tends to be limited at high temperatures, as the layer can interact with and react with SiC [4,5,6]:

SiC (s) + 2 SiO_2_ (s) → 3 SiO (g) + CO (g) (3)

During passive oxidation, the silica film grows, and an increase of mass is observed. However, during active oxidation of SiC, the oxygen reaches the bulk material through cracks or due to the failure of the protective layer, and a mass reduction is observed.

The model for oxidation of SiC shows a relationship of passive oxidation occurring generally at low temperature and high partial pressure of O_2._ The contrary is seen for the active oxidation. Only the passive oxidation is explored in the following sections.

### 2.1. The Passive Oxidation Regime

**Figure 2:**
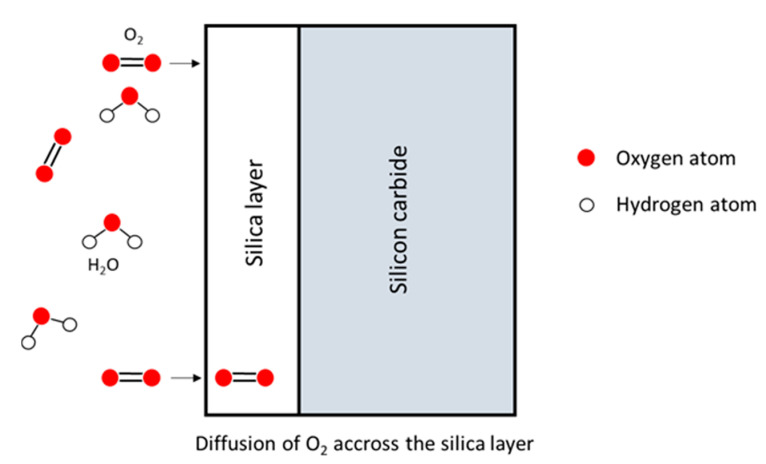
Scheme of diffusion of oxygen.

**Figure 3:**
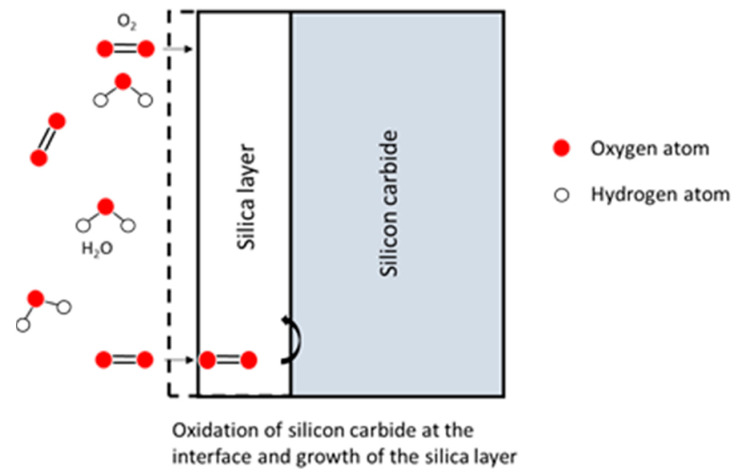
Scheme of the chemical reaction at the interface.

**Figure 4:**
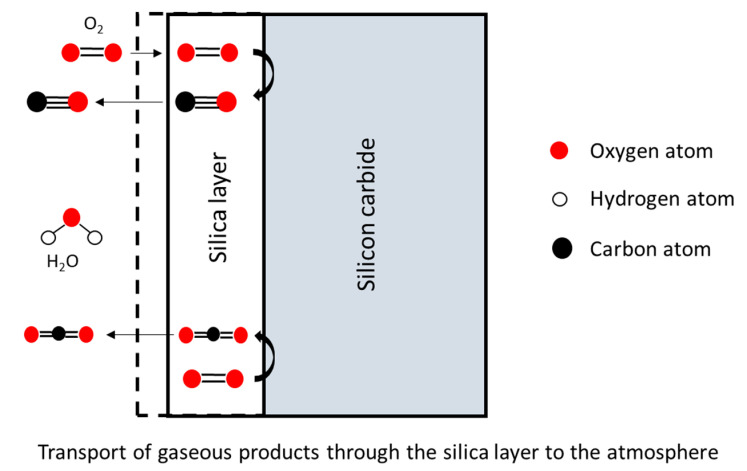
Scheme of the chemical reaction at the interface.

During passive oxidation, the mobile species diffuse through the lattice via cracks or pores. Then, these species react with silicon at the SiO_2_/Si interface or SiO_2_/SiC interface, leading to the growth of the oxide scale [4]:

Jacobson concluded that five mechanisms were involved in the oxidation process of SiC [7]:

1. Transport of molecular oxygen gas to the oxide surface,
2. Diffusion of oxygen through the oxide film (Figure 2),
3. Reaction at the oxide/ceramic interface (Figure 3).
4. Transport of product gases (CO, CO_2_) (Figure 4)
5. Transport of product gases away from the surface.

Between 500 and 800 °C, the oxidation of SiC fibers generates voids and releases gaseous compounds which create a porous silica scale at the SiO_2_/SiC interface. These two facts are probably responsible for the loss of its mechanical properties [8].

Between 1200 and 1400 °C, the silica layer forms rapidly and seals off the surface porosity. The layer tends to delay the inward diffusion of oxygen.

The kinetics of the silica growth depend on the thickness of the oxide layer, which directly relates to the time of oxidation. For thick layers, or a long period of oxidation, the diffusion of oxygen limits the growth of silica, and the kinetics follow a parabolic law. For example, Zheng et al. showed that the parabolic regime of single crystal SiC occurred between 1200 and 1500 °C under pressures from 10^−3^ up to 1 Bar [9]. For thin layers, or a short period of oxidation, the reaction at the interface is the limiting step, and the kinetics follows a linear law. For silica growth which does not follow neither the parabolic nor the linear law, Deal and Grove formulated a linear parabolic law.

Recently, Park et al. used XPS to precisely characterize the chemical nature of the silica layer [10] and discovered that it consists of several oxidation states corresponding to SiO, Si_2_O_3_ and SiO_2_ chemical environments.

### 2.2. Kinetic Models for Si and SiC Oxidation

#### 2.2.1. The Deal and Grove Model

**Figure 5:**
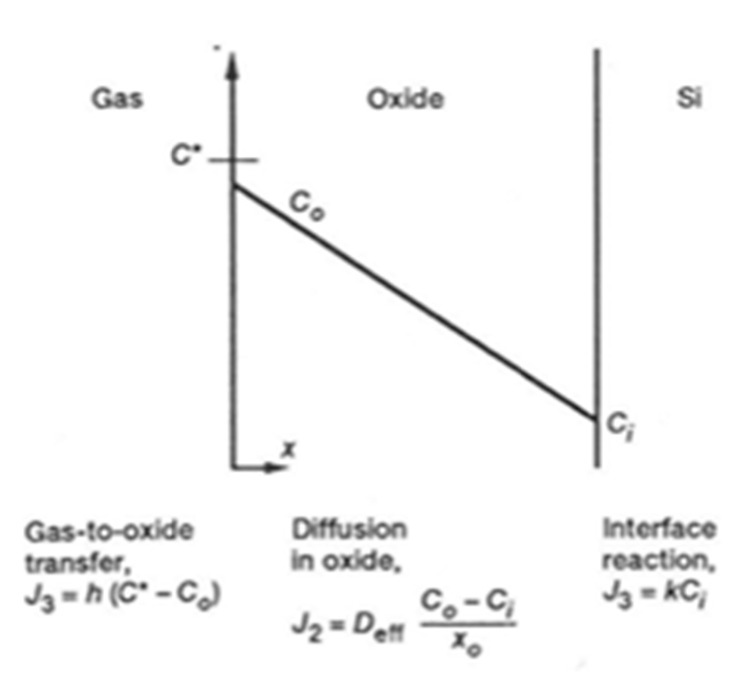
Model for the oxidation of silicon at the two boundaries of the oxide layer. Reprinted with permission from Reference [11]. Copyright 1965 AIP.

In 1965, Deal and Grove developed the kinetic law for the thermal oxidation of silicon (Figure 5) [11] and established a general equation:

(4) $$Ax_{0}=B\left(t+\tau \right)$$

where t is referring to the oxidation time, and the quantity, τ, corresponds to a shift in time).

**Figure 6:**
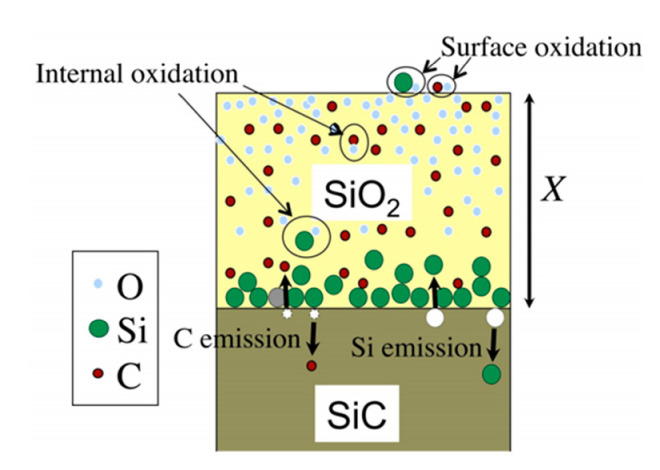
Schematic view of the Si and C emission model. Reprinted with permission from [17].

In this model, both a diffusion process and a model of oxidation are related in Figure 6. The reactions occur at the two boundaries of the oxide layer$\;\left(x_{0}\right)$.

The diffusion of oxidant species through the oxide layer is expressed according to Fick’s first law. Moreover, the model considers the gas phase transport to the oxide and Henry’s law for the interface reaction. The subsequent relations are as follows:

(5) $$B=2D_{eff}C^{*}/N$$

(6) $$C^{*}=KP$$

(7) $$A=2D_{eff}(1/k+1/h)$$

where B is the parabolic rate constant in units of (oxide thickness)^2^/time, $D_{eff}$ is the effective coefficient diffusion of the oxidant species (oxide thickness)^2^/time, $C^{*}$ is the equilibrium concentration of the oxidant in the oxide, N is the number of oxidant species into a volume unit of oxide layer, K is the Henry’s law constant and P the partial pressure of the oxidant. The A parameter is linked to k (reaction rate at the interface) and to the coefficient h (flux of oxidant species entering the oxide).

The Equation (6) can be written as follows:

(8) x0=A21+t+τ/A2/4B1/2−1

At long oxidation times ($t+\tau \gg A^{2}/4B$), a thick oxide layer is created:

(9) $$x_{0}^{2}=B\left(t+\tau \right)$$

This yields the parabolic law of oxidation growth. As B is proportional to $D_{eff}$, the oxide growth is limited by the diffusion of O_2_ through the oxide.

At shorter times ($t+\tau \ll A^{2}/4B$), the Equation (8) can be written as follows:

(10) $$x_{0}=\frac{B}{A}\left(t+\tau \right)$$

where B/A is proportional to the chemical–surface reaction rate constant. Therefore, a linear regime is obtained for the thin oxide formation because the limiting step is controlled by the interface reaction [12].

Thus, Harris et al. were able to correlate this relationship to experimental data obtained over a temperature range of 700–1300 °C, with a partial pressure of 0.1 to 1.0 bar, and for an oxide thickness between 30 and 2000 nm, for both oxygen and water oxidant species.

This model illustrates the passive oxidation of silicon, but multiple studies demonstrated that this oxidation kinetics model fits well with the data obtained for silicon carbide materials as well.

#### 2.2.2. Massoud Empirical Relation

Despite fitting well with most experimental results, the Deal–Grove model is not adapted to the early stages of oxidation (nanometer scale). Indeed, for thickness lower than 1 nm, the oxidation rates are very high and cannot be fitted by linear-parabolic kinetics predicted by the Deal–Grove model. Therefore, Massoud et al. [13] experimentally studied the kinetics of oxidation for very low thicknesses (50 nm). Their hypothesis was that the high oxidation rate can be modeled by adding an exponential term that decays with increasing thickness in the Deal–Grove model.

The modified model is then given by Equation (11):

(11) $$\frac{dX}{dt}=\frac{B}{2X_{ox}+A}+C_{2}e^{-(\frac{X_{ox}}{L_{2}})}$$

This model fits well with data obtained for low thicknesses of oxide, but it is important to emphasize that it is an empirical solution that does not precisely take into account the physical mechanisms occurring during the early stages of oxidation [14].

Until very recently, no model could unify the Deal–Grove model with a model that takes into account both the early stages of oxidation and the physical mechanisms associated with them.

#### 2.2.3. Si and C Emission Model

Goto et al. [15] developed a model based on the emission of Si and C atoms during the oxidation. It is a model based on the Si atoms emission model that had been previously developed [16]. The main difference between this model with the Deal–Grove model is that additional mechanisms for oxide growth surface are considered. Contrary to the D–G model that only considers the formation of oxide at the Si-oxide interface, this model considers the emission of Si atoms that can form oxide by two different ways:

- If the oxide layer is thin enough, the Si atoms can diffuse through it and instantly react with the oxidant atmosphere,
- The Si atoms can also encounter oxidant molecules in the oxide layer itself, and react with it.

The growth rate is then obtained by summing the 3 contributions to oxide formation:

(12) $$N_{0}\frac{dX}{dt}=kC_{O}^{1}\left(1-\nu_{Si}\right)+\int_{0}^{X}\kappa {\left(C_{O}\right)}^{2}C_{Si}dx+\eta {\left(C_{O}^{S}\right)}^{2}C_{Si}^{S}$$

With *ν* the emission ratio, *κ* the oxidation rate of Si inside SiO_2_, *η* is the oxidation rate of Si on the oxide surface, and superscript *S* is related to the position of the atom in the oxide layer.

Goto et al. thus modified the Si atom emission model to apply it to SiC materials. A schematic view of the model is given in Figure 6.

Using the work of Kageshima et al. the interfacial reaction rate is defined by the following:

(13) $$k=k_{0}(1-\frac{C_{Si}^{I}}{C_{Si}^{0}})(1-\frac{C_{C}^{I}}{C_{C}^{0}})$$

where $C_{x}^{I}$ and $C_{x}^{0}$ are the concentration of the species at the interface and the solubility limit of the species, respectively.

By modifying the diffusion equations of the Si emission model, diffusions for the reactive species are given as follows:

(14) $$\frac{\partial C_{Si}}{\partial t}=\frac{\partial }{\partial x}\left(D_{Si}\frac{\partial C_{Si}}{\partial x}\right)-R_{1}-R_{2}$$

with

(15) $$R_{1}=\eta C_{O}^{S}C_{Si}^{S}\;and\;R_{2}=\kappa_{1}C_{Si}C_{O}+\kappa_{2}C_{Si}{\left(C_{O}\right)}^{2}$$

(16) $$\frac{\partial C_{C}}{\partial t}=\frac{\partial }{\partial x}\left(D_{C}\frac{\partial C_{C}}{\partial x}\right)-R_{1}^{\prime }-R_{2}^{\prime }$$

with

(17) $$R_{1}^{\prime }=\eta^{\prime }C_{O}^{S}C_{C}^{S}\;\;and\;R_{2}^{\prime }=\kappa_{1}^{\prime }C_{C}C_{O}+\kappa_{2}^{\prime }C_{C}{\left(C_{O}\right)}^{2}\;$$

(18) $$\frac{\partial C_{O}}{\partial t}=\frac{\partial }{\partial x}\left(D_{O}\frac{\partial C_{O}}{\partial x}\right)-R_{1}-R_{2}-R_{1}^{\prime }-R_{2}^{\prime }-R_{3}$$

with

(19) $$R_{3}=h\left(C_{O}^{S}-C_{O}^{0}\right)$$

By numerically solving these equations and using experimental obtained values, it is possible to use Equation (14) to determine the oxide growth rate.

**Figure 7:**
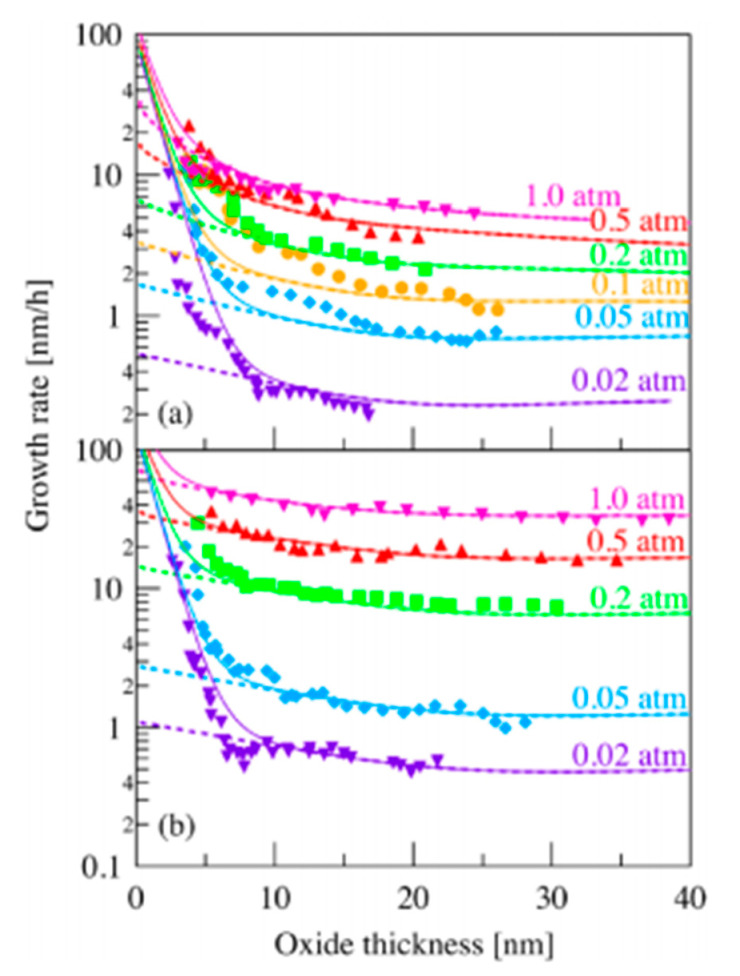
Comparison between experimental results and Si/C emission model for 1100 °C oxidation: (**a**) Si-face and (**b**) C-face. Reprinted with permission from Reference [18]. Copyright 2019 Elsevier.

As an example, results are given on Figure 7 for a fixed temperature and different partial pressures of oxygen for the C-face and the Si-face [18].

The results given here show notable similarity between the model and the experimental results. This validates the model even for very small thicknesses of silicon oxide.

In the next section, the parameters influencing the oxidation of SiC materials are discussed. It is important to point out that the discussion is based on studies using the Deal–Grove model, as it is still the most used model to describe SiC oxidation. Recent studies on the parameters influencing SiC oxidation and using the Si and C emission model include several references [19,20,21,22,23,24].

### 2.3. Parameters Which Can Influence the Oxidation of SiC Materials

Numerous authors calculated the activation energy $E_{A}\;$of the parabolic oxidation of SiC thanks to Arrhénius Equation (9):

(20) Xn=Cte−EART

where C is a constant, X is the oxide thickness, t is the oxidation time and T is the temperature. The activation energy corresponds to the minimum energy required for a chemical reaction to occur. The data for the activation energy for linear oxidation and for parabolic oxidation are shown below, in Table 1 and Table 2:

* Please note that linear parabolic refers to the case when both linear and parabolic regimes are observed.

#### 2.3.1. Interpretation of the Activation Energy Values

The first data on the oxidation of SiC powders are all in the same order of magnitude, lying between 85 and 209 kJ/mol [11,35,36,37,38]. According to the authors, four remarks can be made:

1. Their data differ depending on the fitting of experimental values and the nature of SiC samples,
2. Their data differ due to the presence of impurities from either the sample or the apparatus, or the gas phases present. Thus, the oxidation rate is determined by the nature and concentration of impurities as well as other physicochemical parameters,
3. The oxidation period seems to affect the oxidation kinetics:
  ➢ For short oxidation times, a thin amorphous oxide film is created, and the kinetics of the oxide growth follow a linear regime, which implies that this mechanism is surface-controlled,
  ➢ For long oxidation times, the oxidation rates decrease as the oxide layer grows. The kinetics follow a parabolic regime, meaning that the mechanism proceeds by gas diffusion through the oxide layer [25,28],
  ➢ An initial period of sixty minutes is observed between 1100 and 1300 °C for which the parabolic law fits the data well. Then, at short oxidation times, the silica growth follows a linear regime with an increase of oxidation rates, meaning that the silica scale loses its protective effect [25,35].
4. Finally, the rate determining step of the oxidation is thought to be either the inward diffusion of oxygen or the outward diffusion of CO (i.e., product gases).

First, Deal and Grove established a kinetic model for oxidation of silicon under wet and dry atmospheres [11]. They defined a parabolic constant, which expresses a diffusion-controlled mechanism, and a linear constant, which expresses a surface-controlled mechanism. As the parabolic activation energy of the silicon oxidation is close to the value of oxygen permeation through fused silica (113 kJ/mol)—given by Norton [43] from the literature data—Motzfeldt concluded that oxidation rates of silicon and silicon carbides were similar [44]. It can be concluded that, in both cases, the diffusion of oxygen controls the oxidation kinetics. However, the initial period of SiC oxidation was not implemented into the Deal and Grove model.

Secondly, Jorgensen et al. proposed that the oxidation rate decrease could be due to the crystallization of the scale over long oxidation times [37]. Amorphous silica is produced by the oxidation reaction and can be transformed into cristobalite above 1200 °C, that slows down the diffusion of oxygen slows down.

Finally, the next section deals with the effect of impurities on SiC oxidation and the oxidation time dependence of silica growth.

#### 2.3.2. Nature of the Silica Layer

**Figure 8:**
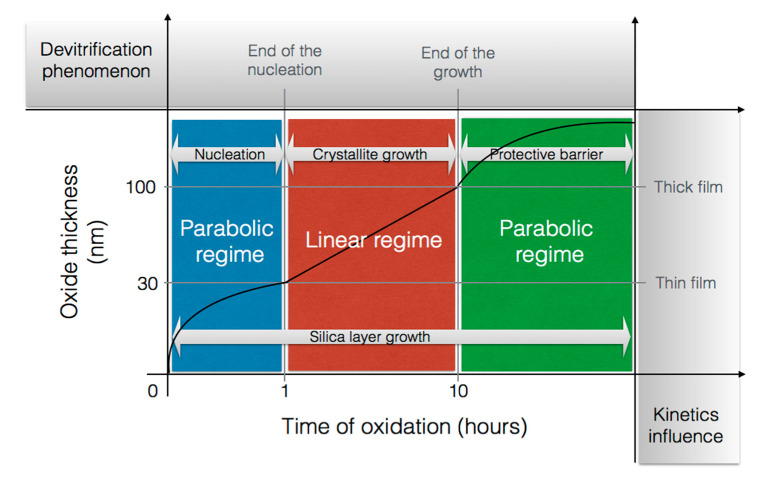
Representation of the kinetics of silica growth and the transition phases of amorphous silica versus time of oxidation in the temperature range of 1000–1300 °C.

Jorgensen et al. [37,45] claimed that, for low temperatures, the silica layer was mainly consisted of amorphous silica, but at higher temperatures and/or after long periods of time, crystallization occurred. Costello et al. confirmed that the activation energy increases with the temperature and/or with the oxidation time. The low activation energy values (134 and 155 kJ/mol) could be attributed to the diffusion of molecular oxygen through an amorphous scale, whereas crystallization of silica could occur at high temperature. This is likely why the highest values (398 and 498 kJ/mol) are recorded [29]. Thus, the transport of oxygen through crystalline scales is thought to be slower than through amorphous [31], as the oxidation rates decreased by a factor of thirty when the scale crystallization was completed [33]. A representation of the phase transitions of amorphous silica layer with time during the SiC oxidation are related on the scheme below (Figure 8):

The purpose of this scheme is to show that the devitrification of silica is in competition with the growth of the oxide layer during the nucleation period and the crystallite growth. However, when the scale is fully crystalline, silica growth is the only mechanism driving the kinetics.

As it can be seen, there is an initial period which seems to correspond with the nucleation of the cristobalite crystals within the amorphous scale. Deal and Grove did not consider this initial period in their model because it was not possible to measure such low thicknesses. However, they suggested the existence of another oxidation regime when the oxide thickness was below 30 nm. At very short oxidation times, silica growth follows a parabolic regime controlled by oxygen diffusion. Then it follows a linear regime controlled by a surface reaction. Finally, it returns to a parabolic regime at long oxidation times. It seems that two processes compete, one increasing the protective property of the oxide scale and the other one degrading it.

At the end of the crystal nucleation, the protective property of the scale is not maintained anymore, as the cristobalite growth generates numerous defects through which speed up oxygen diffusion [46]. Thus, in this second stage, the oxidation mechanism follows linear kinetics both because the silica layer does not limit the diffusion of oxygen anymore and because the crystallization rate is linear under dry atmospheres [47].

For the first stage, devitrification of silica has already occurred, but the layer is still protective. The hypothesis could be that the impurities, which act as crystallization starting point, induce a local decrease of the oxide viscosity. The consequence is a decrease of interfacial stress, which limits the crystallite growth and, therefore, retards the devitrification process. This hypothesis is supported by the work of Wei and Halloran who demonstrated that the devitrification of silica into cristobalite can be avoided by adding mullite grains (i.e., impurities) into vitreous silica [48]. In this first stage, the scale is still protective as the oxygen diffusion proceeds through the amorphous scale; however, it proceeds via cracks and pores as the crystal grows. At this point, defects allow a fast diffusion of oxygen through the scale, and the kinetics follow a linear regime.

Finally, a second hypothesis can be proposed: the impurities can affect the oxidation behavior of polycrystalline material by forming either high or low protective films, depending on the impurity’s concentration. For low concentrations, the silica growth will show an initial oxidation period, and at the end of the second stage, a crystalline layer is formed with low permeability. However, for high concentrations, a rapid initial growth rate is observed, which decreases with the crystal sizes [47]. In this case, the crystal growth is limited, and a partially crystalline layer is obtained. The consequence is that the silica scale shows a high level of microporosity, allowing fast diffusion of oxygen.

This second hypothesis is supported by the work of Costello et al. who demonstrated that a high level of impurities and nucleation sites in SiC materials led to a greater susceptibility to crystallization of the scale and complicated the oxidation behavior [30]. It was found that the presence of cations, impurities and additives (such as aluminum or carbon atoms) led to the formation of a viscous layer with high permeability to oxygen, which was responsible for the increase of the oxidation rates. The crystallization of the scale can lead either to low oxidation rates—when a continuous layer of spherulitic crystals (cristobalite) is formed—or to high oxidation rates—when these crystals are randomly dispersed in the amorphous matrix and locally increase grain boundaries [46].

#### 2.3.3. Crystal Faces Effects

**Figure 9:**
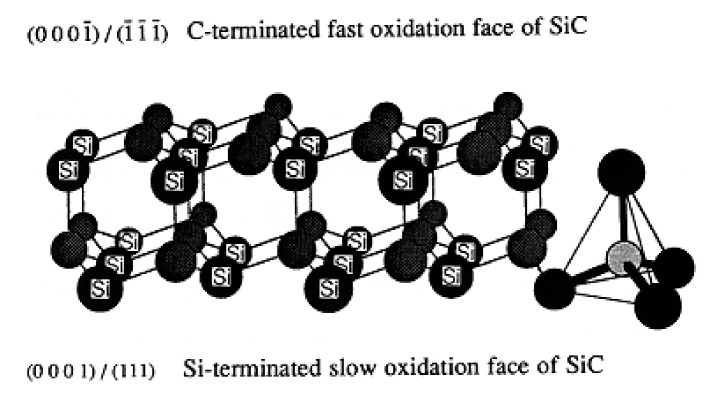
Schematic showing different terminations of the (000$\bar{1}$)/($\bar{1}$$\bar{1}$$\bar{1}$) and (0001)/(111) faces of SiC. Stacking in this case is for cubic SiC. A representative Csi4 tetrahedron is also shown. Reprinted with permission from Reference [27]. Copyright 1996 The American Ceramic Society/Wiley.

In the silicon carbide structure, one carbon atom is linked to 4 atoms of silicon, forming CSi_4_ at their vertices. Double layers of atom are formed exhibiting one carbon face (000$\bar{1}$/$\bar{1}$$\bar{1}$$\bar{1}$) and one silicon face (0001)/(111) referenced as C-face and Si-face (Figure 9) [27].

**Figure 10:**
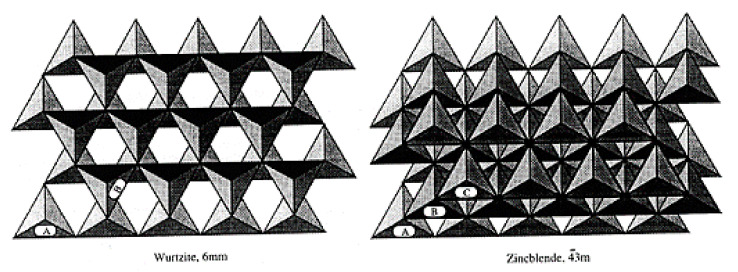
Tetrahedral representation of close-packed planes in cubic and hexagonal SiC. Top schematic shows Zincblende (cubic) structure with CSi4 tetrahedra stacking in ABC periodicity; bottom schematic shows Wurtzite (hexagonal) structure with tetrahedral stacking in AB periodicity. Reprinted with permission from Reference [27]. Copyright 1996 The American Ceramic Society/Wiley.

The network is predominantly covalent and exhibits two general crystalline forms: the cubic β-SiC and the α-SiC. These networks have different crystallographic polytypes depending on the stacking of the tetrahedral bilayers. The main polytypes are the cubic 3C (ABC) with a Zincblende crystal structure and the hexagonal (4H for ABCB or 6H for ABCACB), with a Wurtzite crystal structure (Figure 10).

Furthermore, it is interesting to note that single crystals of SiC are often hexagonal (α) and that CVD-SiC samples generally crystallize in the cubic (β) crystalline. The different crystal structures and the different atomic natures of the C- and Si-faces lead to different oxidation behaviors which is not the case for silicon crystals [49].

The oxidation behavior of the (110, 111, 311, 511 and 100) faces of silicon single crystals

Lewis and Irene obtained different oxidation rates for the thermal oxidation of single crystal Si depending on the crystal faces (110, 111, 311, 511 and 100). It appears that, for thin oxide film, the growth of silica depends on the density of atoms, which is specific to each orientation. Although the oxidation rates increase with the silicon atom density, the development of intrinsic oxide stress becomes dominant for thick oxide films. This affects the transport of oxidant species to the interface and leads to a decrease of oxidation rates below 1100 °C [49]. The influence of the C- and Si-face on the oxidation rates is discussed below.

The oxidation behavior of the slow and fast oxidation faces.

**Figure 11:**
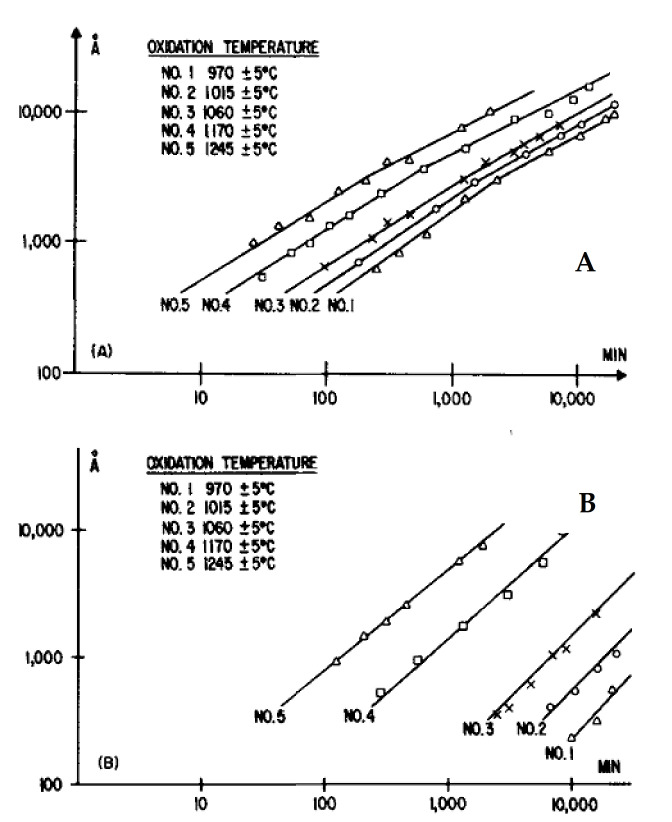
Oxide thickness vs. oxidation time on (**A**) thick oxide side and (**B**) thin oxide side. Reprinted with permission from Reference [12]. Copyright 1974 The American Ceramic Society/Wiley.

Harris first demonstrated that the oxidation of the two crystal faces of SiC platelets followed different kinetic laws in accordance with the Deal and Grove model [12]. Between 1000 and 1300 °C, the C-face showed faster oxidation than the Si-face. The oxidation of the C-face follows parabolic law and leads to the formation of a thick oxide layer whereas the oxidation of the Si-face follows linear kinetics and a thin oxide layer is created, as seen in Figure 11 below.

Many authors confirmed that the oxidation behavior of the fast oxidation face (C-face) of CVD-SiC and single-crystal SiC is similar to that of single-crystal silicon over the temperature range of 1200–1400 °C [9,12,25,27,30,50]. The slow oxidation faces (Si-face) of CVD-SiC and single-crystal SiC also exhibit similar oxidation behavior and activation energy. Firstly, the activation energy is identical for silicon and C-faces of silicon carbide materials, and the crystalline structure of SiC does not seem to have any on its value. Secondly, a change in the chemical composition of the oxide scale is noted [27] which gives evidence for different diffusional processes. An inner layer of unknown composition was found at the SiC/SiO_2_ interface of the Si-slow oxidation face. This layer had higher refractive index and may have had lower permeability, which could explain the change in activation energy. Later, XPS analysis revealed a C-rich region in the oxide scale in the form of silicon oxycarbides that had been formed by the reaction of silica with the carbonaceous species released via Equations (1–3) [51]. The presence of C-rich regions was revealed, as well, by EELS, and these C-rich regions took the form of carbon clusters with a thickness of 10–15 Å [52].

Harris demonstrated that the oxidation rate of the C-face was increasing when temperatures and oxidation time periods were decreasing.

The oxidation behavior of the C-face depending on temperature:

The same observations were made by Zheng et al. [9]: two different activation energies for the oxidation of the C-face were recorded between 1200 and 1500 °C, but only one was recorded for the Si-face of single crystal SiC. The explanation could be that two oxidation kinetics are competing—one dominant at high temperatures and for long oxidation periods, and the other one being dominant at low temperatures. In fact, if the temperature and the oxidation time periods increase, the linear oxidation rate of the material will become dominant over the parabolic law, and the oxidation rate will increase [12]. This implies that the growth of the oxide is surface-reaction controlled and not oxide-diffusion controlled.

Another explanation is a change in the diffusion mechanism of the oxidant species. Costello et al. submitted the idea that if lattice diffusion occurs [30], high activation energy (400 kJ/mol) will be recorded in comparison to a classic oxygen permeation, which is associated to a low activation energy (of 120 kJ/mol).

Zengh et al. agreed and demonstrated that the activation energy was influenced by the chemical change of the diffusing species. Below 1350 °C, a low activation energy value is obtained, and the major diffusing species are molecular oxygen. However, above 1350 °C, a high activation energy occurs, which seems to be the consequence of ionic diffusion [9]. Nevertheless, Ogbuji et al. recorded only one activation energy during parabolic oxidation over the range 1200–1500 °C, which was equal to the value obtained by Deal and Grove [33]. The experiments were performed under highly dry oxygen with clean samples and apparatus, so the conclusion was that only the permeation of oxygen through the scale was limiting the oxidation of SiC, up to 1500 °C. A review on the growth of silica during the oxidation of SiC details these observations [53].

#### 2.3.4. Oxidation Rate-Determining Step

Initially, Pultz and Singhal [28], recorded high activation energies and assumed that the oxygen transport was not the rate controlling step of the oxidation. Singhal concluded that it might be the desorption of volatile carbonaceous products released at the SiC/SiO_2_ interface. However, Antill et al. [42] revealed that the pressure of CO_2_ had no influence on the kinetics over the range 0.2–1 bar, so the pressure was not controlling the reaction. Moreover, he assumed that the diffusion of carbonaceous species through the silica layer did not affect the layer’s protective property, as silicon and silicon carbide demonstrated similar reactivity between 1200 and 1300 °C.

**Figure 12:**
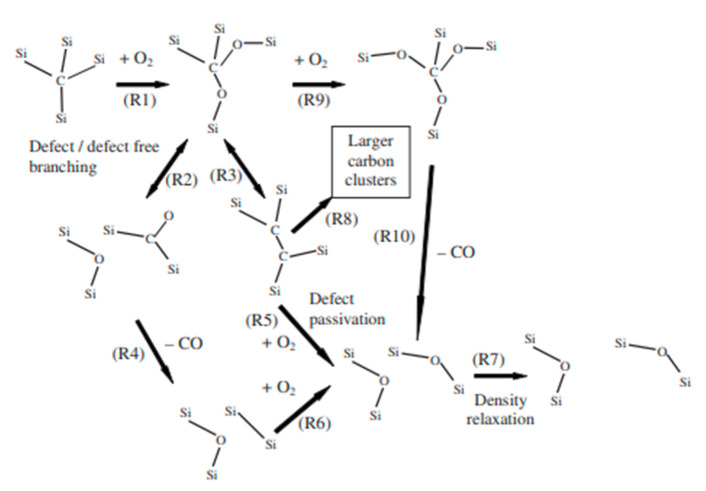
The scheme of the kinetic mechanism of the (0001) SiC surface oxidation process. Reprinted with permission from Reference [54]. Copyright 2008 AIP Publishing.

Nevertheless, the discovery of some C-rich clusters at the SiC/SiO_2_ interface may suggest that the trapping of carbonaceous species (CO or CO_2_) could limit the diffusion out into the gas phase, and, thus, limit the whole oxidation process. However, Zheng et al. established the profile concentration of C18O molecules and demonstrated that the concentration was constant through the silica scale [9]. This confirms fast carbon transport out of the silica scale. The carbon diffusion was also not dependent on the oxygen partial pressure, whereas the oxidation rates of SiC increased with the oxygen pressure. Thus, the transport of carbonaceous species could not be the rate-controlling step of the oxidation. As suggested by Narushima et al., it is possible that the diffusion of oxygen ions into the silica network is why high activation energy values are observed [31] Gavrikov et al. investigated the defect generation and passivation of the Si-face of SiC dry oxidation [54]. An abrupt model which describes the transition between crystalline SiC and amorphous SiO_2_ was used to perform calculations of SiC oxidation reactions. Thus, the mechanism kinetic was designed for a rigid silica scale (i.e., at temperatures above 1300 °C and without water hydration), as described below (Figure 12):

The mechanism described above can be explained within four steps:

1. The penetration of oxygen into the silica scale:

First, the transport of oxygen at the Si-face is considered (R1, on Figure 12). The calculated activation energy is 250 kJ/mol for molecular diffusion of oxygen through the oxide layer.

However, the oxygen diffusion can proceed through lattice oxygen atoms with higher calculated activation energies. The value is about 390 kJ/mol, and therefore the lattice diffusion of oxygen is not considered to contribute to the SiC oxidation.

2. Reaction of oxygen with SiC:

The oxidized bonds Si-O-C are created at the SiC/SiO_2_ interface and three chemical reaction paths are observed:

The first one consists of the formation of a carbonyl defect: Si_2_ = CO (R2, on Figure 12), which is followed by CO desorption (R4, on the Figure 12). DFT calculations predict a high activation energy of 350 kJ/mol, but this path proceeds faster at high temperatures. Then, further oxidation creates new silica units (R6, on the Figure 12) with an activation energy of 190 kJ/mol.

The second path consists of the formation of C_2_ dimer intermediates: Si_2_ = C = C = Si_2_ (R3, on Figure 12), which evolve into larger aggregates (R8, on the Figure 12). This creates carbon defects near the interface.

The last path consists of further oxidation of the Si-O-C bonds (R9, on the Figure 12) followed by CO desorption (R10, on the Figure 12). DFT calculations show that three oxidized bonds are necessary for CO desorption, and that the activation energy is 190 kJ/mol.

3. Passivation reactions of carbon defect by O_2_:

Calculations show that the carbon defect could react with an oxygen molecule in singlet state to give new silica entities: Si_2_ = C = C = Si_2_ + O_2_ → Si_2_ = C = C = O + O = Si_2_ (R5, on Figure 12). The activation energy for this is 190 kJ/mol.

For temperatures above 900 °C, the calculations shown that the reaction rate of carbon with an oxygen in a triplet state is higher. An activation energy of 60 kJ/mol is calculated for the dissociation of the oxygen molecule: Si_2_ = C = C = Si_2_ + O_2_ → Si_2_ = C = C(Si)-O-Si + O

4. Density relaxation process:

The new silica units undergo structure relaxation (R7, on Figure 12) in order to decrease the bond density at the interface and thus lower the stress energy. The activation energy is 190 kJ/mol.

The interest of this model is to underline the complexity of the oxidation mechanism of SiC. As it is described, ten reactions are involved in the oxidation process, but only one can control the silica growth kinetics. Gavrikov et al. used an abrupt model for the SiC/SiO_2_ interface, in which a high activation energy was predicted for the oxygen penetration. Thus, they assumed that oxygen diffusion (R1) was the rate-determining step of the oxidation process, whereas CO desorption was competing with carbon defect generation.

### 2.4. Conclusions

The oxidation of SiC materials can be either passive or active, leading to the formation of a silica layer in the passive case, and leading to oxide gaseous compounds in the active case. From a general point of view, the oxidation of these materials leads to the degradation of SiC and to the loss of its mechanical properties. For high-performance applications, it is necessary to better understand this phenomenon, which is why kinetics model for the passivation of silicon are studied example, Deal and Grove elected a linear parabolic regime for the oxidation of silicon under dry and wet atmospheres.

Different parameters can influence the oxidation behavior of SiC materials, and, thus the kinetics:

- At short oxidation times, a gas diffusion mechanism is dominant (parabolic regime) whereas at long times, a surface-reaction mechanism is dominant (linear regime),
- For the gas diffusion mechanism, the temperature plays an important role: at low temperatures, the oxygen diffusion is molecular, whereas above 1350 °C, the diffusing species is ionic oxygen. Therefore, a C-rich inner oxide layer is created on the Si-faces,
- The oxidation behavior becomes complicated when the crystallization of amorphous silica takes part in the oxidation process. This reduces the oxidant transport and leads to the decrease of oxidation rates.
- Finally, the presence of impurities is not negligible and may have an impact in all these studies. A high degree of impurities will enhance both the crystallization of the scale and the formation of defects, which allows for faster oxygen diffusion.

From these points, researchers started to investigate the oxidation mechanism of SiC to explain the differences in activation energy values.

**Figure 13:**
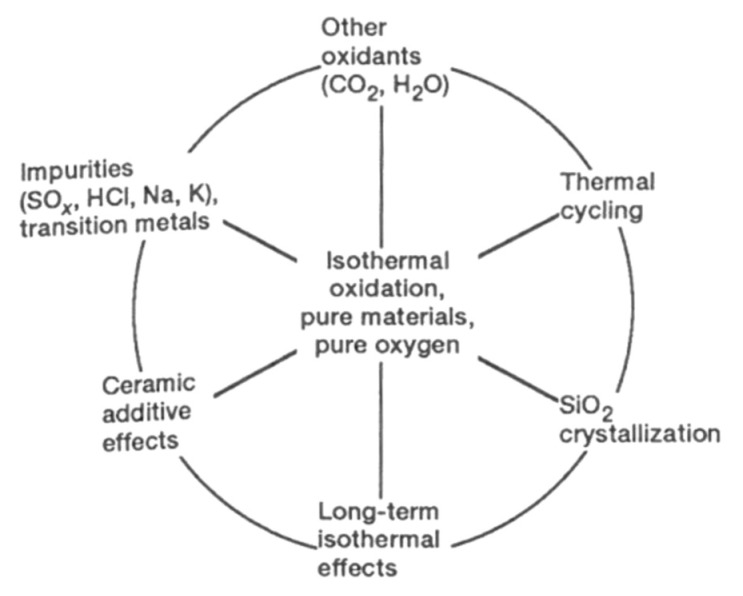
Complications to isothermal oxidation due to additive-containing ceramics and combustion environments. Reprinted with permission from Reference [7]. Copyright 1993 The American Ceramic Society/Wiley.

A large amount of research has been performed on the isothermal, passive oxidation of silicon-based ceramics. Jacobson [7] highlighted that the combination of various secondary effects (on the outer circle below) and the complexity of the combustion environments makes the oxidation process difficult to understand. This directed fundamental studies to focus on the center of the circle—pure materials and pure oxygen environments (Figure 13).

As shown, the SiC oxidation is influenced by both numerous operating parameters and by intrinsic material properties. In the following part, the effect of water molecules on the oxidation of SiC materials is reviewed.

## 3. Wet Oxidation of Silicon Carbide Materials

**Figure 14:**
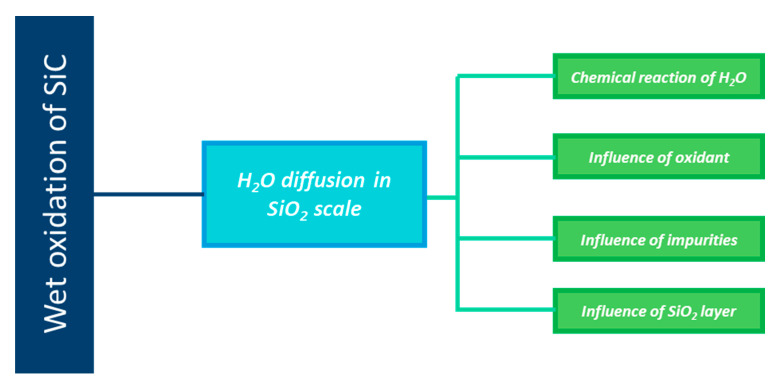
Layout of the wet oxidation part of the review.

Figure 14 gives a scheme describing the layout of the next section.

Nakatogawa [35], Suzuki [55] and later Jorgensen et al. [38,46] studied the effects of water vapor on the oxidation of silicon carbide powders. Jorgensen et al. found out that the presence of water vapor in O_2_ accelerated the oxidation rate of SiC and the nucleation rate of cristobalite.

The interaction of water with the SiC material produces a silica layer which can be described by the following hydrothermal oxidation reaction 21:

SiC (s) + 3 H_2_O (g) → SiO_2_ (s) + CO(g) + 3H_2_ (g) (21)

However, some authors have proposed that Equation (22) competes with (21) [56]:

SiC (s) + 4H_2_O (g) → SiO_2_ (s) + CO_2_ (g) + 4H_2_ (g) (22)

Chaudhry used Auger Electron Spectroscopy to assess the chemical composition of silica films grown on SiC materials under dry and wet oxygen. The content of carbon atoms within the film was higher for wet oxidation (14 at%) than for dry oxidation (2 at%). Following the (19) equation, CO_2_ molecules are produced at the interface. As the diameter of CO_2_ (3.0 Å) is bigger than the ring width of SiO_2_ (2.5 Å) [57], the molecule is easily trapped within the growing scale.

Furthermore, the high content of carbon can result from the high reaction rates of water with SiC, which then releases a large amount of carbonaceous species at the SiC/SiO_2_ interface. Due to this, the silica layer catches more volatile species. The higher permeation of water, as opposed to the permeation of oxygen, through the oxide scale explains the high oxidation rates [46,58]. A new oxidation regime is described in the next section in order to explain the fast kinetics of SiC wet oxidation.

### 3.1. The Two Competitive Oxidation Regimes

Two regimes of oxidation compete depending on the operating parameters.

The passive oxidation regime is described by Deal and Grove using linear parabolic kinetics for the wet and dry oxidation of silicon [11]. From this model, the calculated permeation of oxygen through silica was one thousand times less than that of water. In other words, a water content in oxygen gas of less than 25 ppm will affect both the surface kinetics during linear oxidation and the diffusional kinetics during the parabolic oxidation [59].

*The mixed oxidation regime* is described by Rosner et al. and Opila using Tedmon’s treatment of paralinear kinetics for Cr and Fe-Cr alloys [59]. In this model, both diffusive and gas/oxide interface processes occur simultaneously, and the oxide scale grows to a limiting thickness.

During the oxidation of CVD-SiC in a 50% H_2_O/O_2_ gas mixture, between 1200 and 1400 °C, the two oxidation reactions are (23) and (24) [42,60,61,62,63,64]:

SiC (s) + 3H_2_O (g) → SiO_2_ (s) + 3H_2_ (g) + CO (g) (23)

SiC (s) + 2H_2_O (g) → Si(OH)_4_ (g) (24)

The rate of reaction (22) is described by the parabolic rate constant for oxide formation $k_{p}$, whereas that of reaction (24) is described by the linear rate constant for oxide volatilization $k_{1}$. The evolution of the oxide thickness x with the time t is described by the following relation:

(25) $$\frac{dx}{dt}=\frac{k_{p}}{2x}-k_{1}$$

Over long periods of time or high volatility rates, the rate for oxide formation equals the rate for oxide volatilization, and a steady state is reached. At that moment, SiC undergoes a linear recession given by the rate $y_{L}$ which is proportional to the volatility rate of the oxide, $k_{1}$. The oxide thickness is reaching a maximum, $x_{L}$:

(26) $$x_{L}=\frac{k_{p}}{2k_{1}}$$

**Figure 15:**
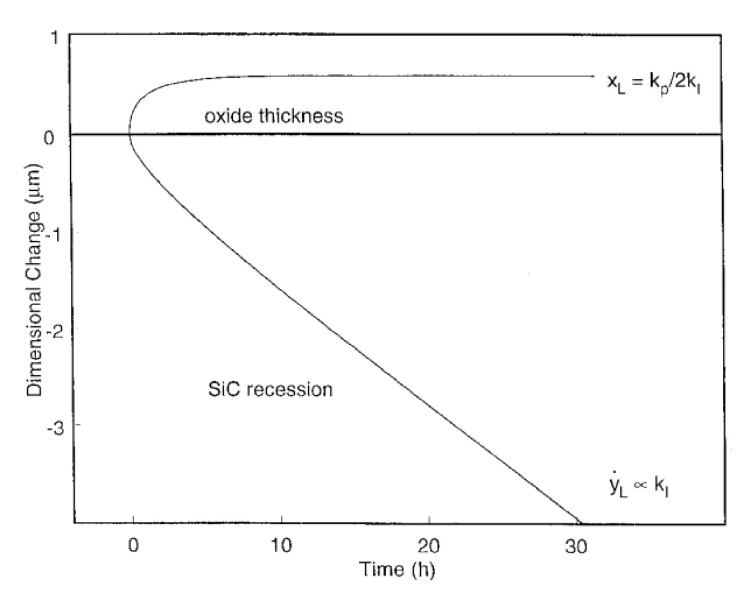
Dimensional change for SiC due to paralinear oxidation and volatilization. Oxide thickness and SiC recession curves calculated for $k_{p}$ = 0.25 mil^2^/h and $k_{1}$ = 0.21 mil/h (1 mil = 1 × 10^−3^ in. = 25.4 µm). Reprinted with permission from Reference [64]. Copyright 2003 The American Ceramic Society/Wiley.

This relation can be expressed in terms of weight change from the integrated form of Equation (25) and the figure below shows the evolution of dimensional change for SiC with the oxidation time (Figure 15):

The kinetics of Equation (22) are described by the parabolic rate constant for moderately thick scales, in which the rate constant $k_{p}$ is dependent of the partial pressure of water vapor, $P_{H_{2}O}^{n}$ but not of gas velocity (24):

(27) $$k_{p}\propto P_{H_{2}O}^{n}$$

where n is the power-law exponent. A n value of 1 is used, as other studies demonstrated that the oxidation mechanism was controlled by molecular water diffusion through the silica scale [11,65,66]. A reference temperature of 1316 °C was set to establish the oxidation rates under water vapor (25):

(28) $$k_{p,\;1316{}^{\circ}C}=0.44P_{H_{2}O}^{1}$$

**Figure 16:**
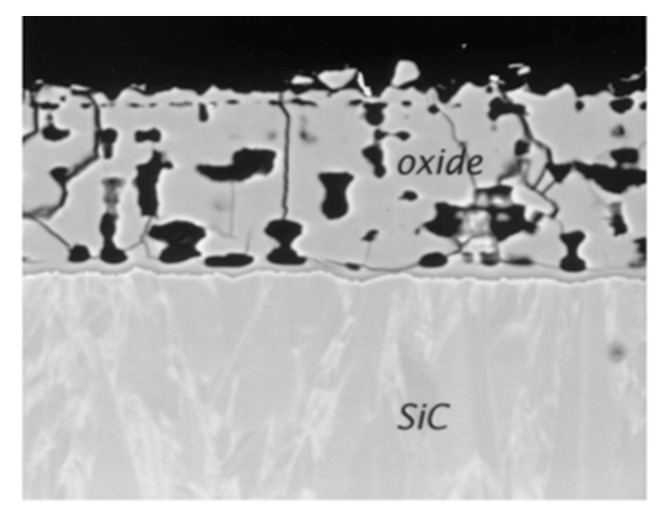
Representative oxide cross-section formed on SiO_2_-formers for the CVD-SiC sample oxidized in a high pressure furnace at 1200 °C and under 10 bar of 15% H_2_O, during 500 h, with a gas velocity of 0.05 cm/s. Reprinted with permission from Reference [64]. Copyright 2003 The American Ceramic Society/Wiley.

One can see that the oxidation rate increases with the partial pressure of water. From reaction (22) it leads to an increase of gaseous compounds at the SiC/SiO_2_ interface responsible for the high porosity of the oxide. This can be seen on the cross-section images of the oxide layer on a CVD-SiC substrate below (Figure 16):

As the oxide layer decreases in density (i.e., increasing in porosity), the gas-phase transport is occurring through the pores. The increase of the distance from the surface to the SiC/SiO_2_ interface does not follow the parabolic law anymore. Thus, the solid-state transport of water is considered to be rate-determining through the dense part of the silica scale. 

To summarize, Opila et al. demonstrated that below 1100 °C, the wet oxidation of SiC follows linear–parabolic kinetics and, above 1100 °C, the oxidation follows simple parabolic kinetics. In the first case, the oxidation is surface-controlled whereas, in the other case, it is diffusion-controlled [64].

**Figure 17:**
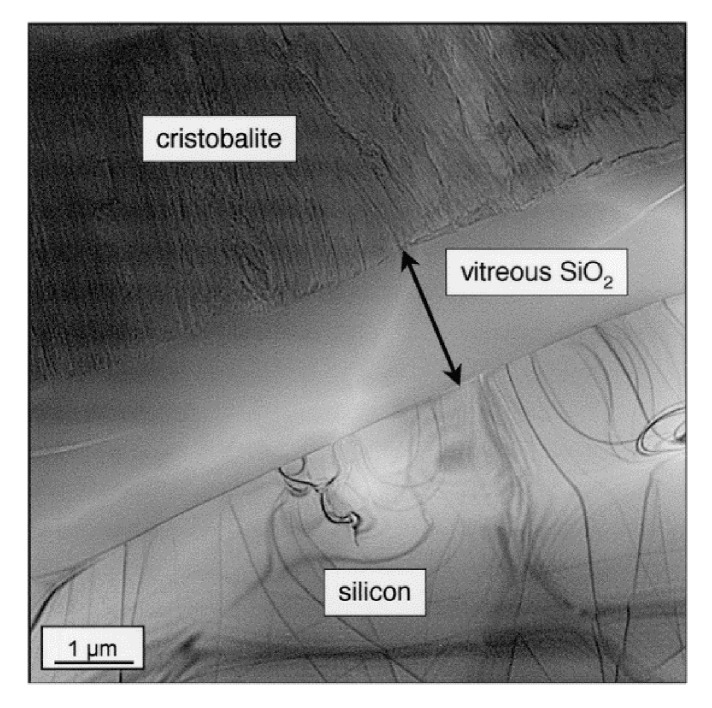
Cross-sectional TEM image of the thin underlying dense layer on pure silicon exposed for 500 h in air + 15 vol% H_2_O at 1200 °C and 10 bar. Reprinted with permission from Reference [67]. Copyright 2003 The American Ceramic Society/Wiley.

Finally, Tortorelli et al. observed the oxide scale after the wet oxidation of pure silicon and CVD-SiC materials [67]. In contradiction with Opila, all the oxide scales were crystalline after a 1200 °C exposure under wet atmosphere [66]. For these samples, a two-layer oxide scale is observed for which the layer at the interface is dense and amorphous, whereas the upper one is thick and crystalline for silicon samples, as shown below on Figure 17.

**Figure 18:**
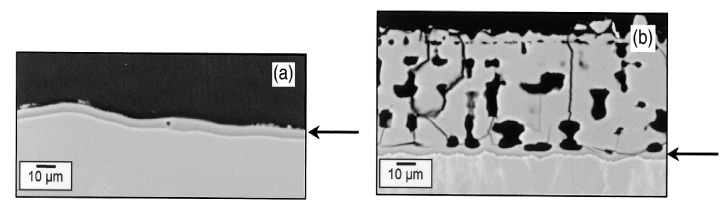
CVD-SiC seal coats after exposures in 1.5 atm H_2_O at the high gas velocity (~30 m/s) of (**a**) Solar Turbines combustor at ~1200 °C for 5016 h and (**b**) low gas velocity (~3 cm/min) of a laboratory furnace (ORNL rig) at 1200 °C after 500 h. Reprinted with permission from Reference [67]. Copyright 2003 The American Ceramic Society/Wiley.

For CVD-SiC samples, carbonaceous species are released by the oxidation under water pressure (1.5 bar). At low speed flow, the volatile species are trapped in the layer, leading to pore formation, whereas at high speed flow, the volatilization of the scale is dominant and no layer remains (Figure 18):

As was observed by Opila [63], the thickness of the thin film was constant with time, whereas the crystalline oxide layer was continually increasing. Therefore, the volatilization of the cristobalite layer is considered not to contribute to the SiC recession. In addition, the high rate of the cristobalite growth could be predicted neither by the paralinear model nor by the presence of impurities. However, the fact that the dense layer does not thicken with time could be the expression of another paralinear regime. In this new model, the thickness of the dense layer becomes constant as the rate of its formation is equal to the rate of its transformation into a porous scale [66]. In the model of Haycock, two density conversion factors are used for relating the thickness of reacted material to the thickness of the dense and the porous scales. Agreement of the parabolic rate constant with the experimental data shows that the paralinear model is valid at high water pressure and low gas-flow velocities.

To conclude, the paralinear model is used depending on the experimental conditions as temperature, pressure, gas velocities and oxidation time. Moreover, Tortorelli et al. demonstrate that the transformation from a dense to a porous layer leads to the formation of a non-protective scale. As a consequence, this formation has a great influence on kinetics. The study of the activation energy, nature of the silica scale, and the diffusion of species leads to better understand of the wet oxidation mechanism of SiC.

### 3.2. Main Characteristics of Wet Oxidation of SiC Materials

Numerous authors calculated the activation energy of the oxidation of SiC, for which the values lie between 46 and 428 kJ/mol. However, there are two exceptions of 531/656 and 527 kJ/mol, which were reported by Palmour et al. and Singhal et al., respectively [58,68,69]. The data for linear and parabolic oxidation are reported in the two tables below (Table 3 and Table 4): 

#### 3.2.1. Activation Energy of SiC Wet Oxidation

According to the Deal and Grove model, the parabolic rate expresses a diffusion-controlled mechanism whereas the linear rate expresses a surface-controlled mechanism.

For the linear rate, most of the values are between 109 and 218 kJ/mol, but the authors do not give any explanation for the oxidation mechanism [26,50,57]. High values (around 300 kJ/mol) [71] suggested that the breaking of Si-C bonds (290 kJ/mol) is the rate-controlling step of the linear growth of silica.

Most of the parabolic values are between 100 and 300 kJ/mol, which is comparable with the data obtained for the dry oxidation of SiC. In accordance with Jorgensen et al., Deal and Grove and Singhal et al. [11,46,62] the activation energy of SiC was affected when water vapor was added to oxygen due to particular dissolution of water through the silica layer.

However, low parabolic activation energy of 41 and 68 kJ/mol were recorded by Opila and Deal and Grove [11,32]. The first explanation for such low values is that the experiments were performed under highly controlled-atmosphere with low levels of impurities, and the second is that Opila showed agreement with the Deal and Grove model. However, Opila et al. suggested that only water vapor diffusion could be the rate-determining step of the parabolic growth of silica.

In opposition, Singhal et al. obtained high activation energy (526 kJ/mol), which was close to the activation energy for dry oxygen (481 kJ/mol), and concluded that the impurities could lead to the devitrification of the oxide scale. According to Ainger, water has the capacity to enhance the crystallization of cristobalite [40], leading to cracks and defect formation into the amorphous layer. Therefore, the cracks would allow water, oxygen and impurities to diffuse faster, enhancing the oxidation rates.

#### 3.2.2. Impurities Effect

First, Opila calculated the rate of oxidation under water vapor (10 wt% H_2_O) according to Deal and Grove model of silicon oxidation [32]. The rate of oxidation was one order of magnitude higher when using sapphire tubes, and the activation energy increased from 41 to 249 kJ/mol. This could be a consequence of the transport of alkali ions to the interface induced by the combination of water vapor and impurities. The first hypothesis is that an amorphous sodium aluminosilicate layer is produced. This layer could demonstrate higher permeability to water which could raise the oxidation rate. The second hypothesis is that the presence of alkali ions (aluminum and sodium) allows the nucleation of the silica scale and enhances the transformation of cristobalite to tridymite [75]. Although there are limited data on the permeability of water through cristobalite, due to the conversion to the phase change of silica with temperature, the work of Jorgensen et al. [38], Antill et al. [43] and Lu et al. [25] showed that when tridymite and quartz formed, the oxide layer acted as a protective film to oxidant species. As a consequence, a tridymite layer should have a lower permeability to water than a cristobalite layer, and this shows that the second hypothesis does not seems to be valid.

Nevertheless, the α- and β-tridymite demonstrate the most open crystalline structure in comparison with quartz and cristobalite phases. Indeed, tridymite has more than 50% voids in the unit cell, according to the review paper of Lamkin et al. [76]. The conclusion is that the diffusion of oxygen and water species might be faster through tridymite than through the cristobalite network, and therefore the second hypothesis of Opila appears valid.

To conclude, one can see that the presence of impurities can modify either the permeability of the scale or the diffusion mechanism of oxidant species, leading to a change in the oxidation behavior of SiC materials. The following section deals with the determination of the primary oxidant species during wet oxidation of SiC.

#### 3.2.3. Nature of the Oxidant Species

Jorgensen et al. carried out one experiment to determine the role of the silica nature in the oxidation behavior of SiC under dry and wet atmospheres [46]. First, the wet oxidation of SiC is performed to obtain a tridymite scale at the surface. Then, a second oxidation is performed under oxygen atmosphere. The two oxidation rates are similar; thus, it was concluded that the diffusing species were the same under the partial pressure of water and of oxygen.

In opposition, Opila [66] showed that the presence of oxygen with water vapor plays an important role in the SiC oxidation kinetics. Indeed, the activation energy is found to be inversely dependent on the partial pressure of oxygen [69]. Moreover, Irene and Ghez deduced that the oxidation rate of H_2_O/O_2_ mixtures was greater than the one calculated for simultaneous and isolated oxidation by water and oxygen as primary oxidants [60]. Thus, a kind of synergy is occurring when water is added to oxygen for SiC oxidation. In the next section, the high oxidation rates obtained with water could be related to the nature of the silica scale.

#### 3.2.4. Nature of the Silica Layer

**Figure 19:**
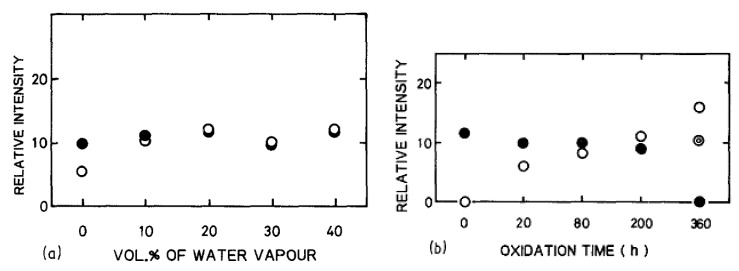
Relative intensities of X-ray diffraction peaks (**a**) against water vapor content (1300 °C, 100 h) and (**b**) against oxidation time (1300 °C, 20 vol% H_2_O), (•) corundum, (o) cristobalite and (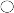) mullite. Reprinted with permission from Reference [74]. Copyright 1988 Springer Nature.

Opila et al. observed an amorphous silica scale after 100 h of oxidation [66] and concluded that water vapor had little effect on the crystallization rate of silica. The analysis of XRD pattern intensity of the oxide growth versus the content of water vapor confirmed this observation [74]. According to Figure 19, the water vapor content does not level up the relative intensity of cristobalite, thus, it does not enhance the crystallization of silica.

As corundum is present in the sintered materials, its peak intensity also appears in the XRD patterns. It was stated in the dry oxidation part that the oxide layer becomes more and more crystalline as the temperature and the oxidation time increase. For wet oxidation, Opila et al. concluded the same by showing that the crystallization is enhanced up to 1400 °C, at which the scale is fully crystalline (i.e., cristobalite) [64]. In addition, Maeda et al. demonstrated that the increase of the cristobalite content in the oxide layer with the oxidation time was faster when 20 vol% of H_2_O was added to air [74]. Therefore, water vapor contributes to the devitrification of amorphous silica but does not increase its protective properties, as high oxidation rates are recorded under wet conditions [33,46,58].

**Figure 20:**
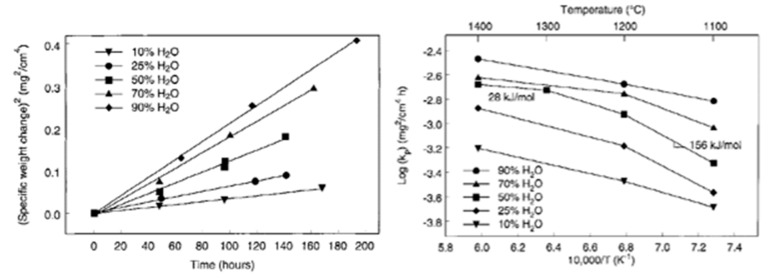
(**Left**)—Determination of parabolic rate constants for CVD SiC in H_2_O/O_2_ mixtures at a total pressure of 1 atm and a temperature of 1200 °C. (**Right**)—Temperature dependence of the parabolic oxidation rate constant for CVD SiC in the temperature range of 1100–1400 °C in various H_2_O/O_2_ mixtures. Reprinted with permission from Reference [66]. Copyright 1999 The American Ceramic Society/Wiley.

In fact, Opila examined the oxidation kinetics of SiC in terms of Deal and Grove model under water/oxygen mixtures [66]. The oxidation rates increased with the water content, but the improvement was not as important as for the lowest contents—therefore the increase of oxidation rate is not linear with the increase of water content, as is shown on Figure 20.

For these experiments, the mix oxidation regime is not considered for temperatures above 1200 °C, as for long times and thick scales, the volatilization is negligible. So, the passive oxidation regime is applied, and, for high water contents, it is shown that the crystallization of the silica scale increases [40] which limits the water diffusion. The Arrhenius plots do not show temperature dependence, which seems to be correlated to a change in either the nature of the oxide scale or in the oxidation mechanism.

Finally, the crystallization rate of silica on silicon was found to follow parabolic kinetics in steam, whereas it obeys a linear regime under dry oxygen [48]. The contradiction is that water species engender higher oxidation rates in comparison with oxygen, but at the same time, the crystallization of the scale is catalyzed. It is known that the solubility of water in oxide is one thousand times higher than that one of oxygen, so high interaction of water with the oxide layer could be the reason for such oxidation behavior.

#### 3.2.5. Particular Reaction of Water with the Oxide Layer

The high reactivity of water is noticed and a new mechanism of water diffusion could be linked to this reactivity [43]. Indeed, Cappelen attributed the high oxidation rates to the high permeation of H_2_O molecules, by incorporation of (HO^-^) into SiO_2_ [70]. Deal and Grove [11] obtained solubility values of water in silica, close to the one of Norton [44], which were one thousand times higher than the solubility of oxygen. The water in contact with silica could react through Equation (29):

Si-O + H_2_O → 2HO^−^ (29)

Based on the Wagner lattice defect model [77], the oxidant diffusion can occur via vacancies in the oxide lattice. If hydroxyl ions are produced, the parabolic rate constant will be proportional to the square root of pressure, as can be seen in the table below, with $H_{2}O_{i}^{x}$ for a water molecule at interstitial site, with $HO_{i}^{\prime }$ and $H_{i}^{˙}$, respectively for hydroxyl ions and protons at interstitial sites, with $O_{i}^{”}\;$for O^2+^ cations at interstitial sites and, finally, with $2h_{i}^{˙}$ for an interstitial site hole. Details of results are given on Table 5.

The fact that n is not exactly equal to 1 could arise from the carrier gases used and from the nature (i.e., chemical composition, porosity) of the scale. For exponents close to 0.5, it seems that the diffusion mechanism involves molecular water and charged species. The fact that the value of the power law exponent is not equal to 0.5 could come from two possibilities:

(1) The dissolution of water produces hydroxyl ions, but not all participate to the growth of the scale [43],
(2) The dissolution of water is not complete, and the diffusive species through silica are molecular water and hydroxyl ions [66].

**Figure 21:**
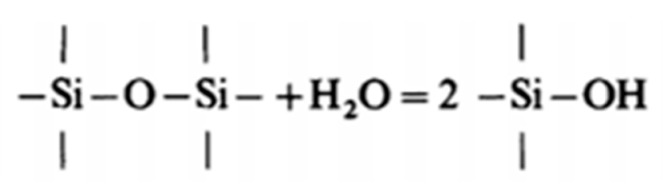
Dissolution of silica during wet oxidation. Reprinted with permission from Reference [68]. Copyright 1981 Acta Chemica Scandinavica, 1947–1999.

Irene and Ghez investigated the second hypothesis [60] and underlined the particular role of water during wet oxidation of single-crystal Si. First, water is the primary oxidant species which allows the conversion of Si to SiO_2_. Secondly, it has the capacity to loosen the SiO_2_ network and thereby increase the diffusion of other oxidant species. The dissolution occurs by reaction to hydroxyl and the breaking of an oxygen bridge, as proposed by Moulson and Roberts [78] and confirmed by Wagstaff [48] (Figure 21):

It was shown that the formation of silanol bonds is reversible, meaning that when the atmosphere is changing from wet to dry, the oxidation rates rapidly regress to that obtained under dry O_2_ [68,71]. Hence, it is necessary to determine the rate-controlling step of the oxidation.

#### 3.2.6. Oxidation Rate-Determining Step

Cappelen [70] showed that the quantity of CO (g) produced during wet oxidation (Equation (22)) is equal to the one produced during dry oxidation. Meanwhile, the oxidation rate under wet atmosphere is ten times higher than that of under dry conditions. As a result, the desorption of carbonaceous species at the SiC/SiO_2_ interface is not the rate-controlling step of silica growth. Later, Narushima et al. [72] obtained a high activation energy value (around 200 kJ/mol) for the parabolic rate constant of wet oxidation. The same value was obtained for the dry oxidation of SiC when a crystalline layer of silica was obtained. This suggests that the rate-controlling step of both diffusion is identical, i.e., the diffusion of O^2−^ ions into the cristobalite film. In the case of wet oxidation, a high number of defects in the layer will be consistent with the O^2−^ fast diffusion through the layer and higher oxidation rates.

To conclude, the last phenomenon to take into account is the crystal orientation effect.

#### 3.2.7. Crystal Orientation Effect

Lu et al. performed dry and wet oxidation on the C-face of SiC in order to create thin oxide film on SiC substrates [25]. It has been stated that the fast-oxidation face (C-face) of CVD-SiC and single-crystal SiC is similar to the one of single-crystal silicon over the temperature range of 1200–1400 °C [9,12,27,30,51]. Though, the oxidation rate of C- and Si-faces was determined to be slower than that of the single-crystal Si (100), depending on the temperature and time of oxidation. Indeed, the C-face rate of oxidation was about 2–4 times slower than the Si (001) material under dry oxygen and about 2–11 times slower under wet oxygen. For both conditions, by increasing the time of oxidation and the temperature, the oxidation rates of the C-face SiC and Si (001) tends to be equal. However, the crystallinity of the scale was not discussed and should be the reason why the oxidation rate of the two faces is not similar.

The most important remark is that similar linear activation energy (about 200 kJ/mol) is obtained for wet and dry oxidations of the C-face. Thus, the oxidation behavior of the C-face of SiC thin films is the same under oxygen or water atmosphere, except that water allows higher reaction rate. To conclude, the rate-controlling step of the linear regime is the same for wet and dry oxidations of SiC materials.

### 3.3. Conclusions

From the parameters which can influence the wet oxidation behavior of SiC materials, the remarks made for the dry oxidation are still valid:

− At short oxidation time, a gas diffusion mechanism is dominant (parabolic regime) whereas at long times, a surface-reaction mechanism is dominant (linear regime),
− For the gas diffusion mechanism, temperature plays an important role: at low temperatures, the oxygen diffusion is molecular whereas above 1350 °C, the diffusing species are ionic oxygen,
− The oxidation behavior is complicated when the crystallization of amorphous silica takes part in the oxidation process. This reduces the oxidant transport and leads to the decrease of the oxidation rates,
− Finally, the presence of impurities is not negligible and could be involved in all these studies. On one hand, it enhances the crystallization of the scale which leads to an increase of defects and, on the other hand, it creates high-permeable and viscous oxides. Both mechanisms result in a faster oxygen diffusion.

Notably, the high reactivity of water has been highlighted, as it has the capacity to enhance the silica devitrification for which high crystallization rates are obtained. Water is able to improve the impurities’ mobility through the network, which leads to the formation of highly permeable oxides.

Finally, the oxidation behavior of SiC is shown to be complex and ambiguous due to its dependence on two parameters: time and temperature. The dissolution and reaction of water with the silica scale seems to be the key to understand the whole oxidation process. When Si-OH groups form, the network relaxes; as a consequence, the SiO_2_ viscosity, density, acoustic velocity and refractive index decrease, whereas the thermal expansion coefficient increases [79,80]. The introduction of water into oxide networks has been modeled by Doremus.

### 3.4. Dissolution-Reaction Model for Water through Oxide Scales

The dissolution models are necessary to understand the interaction mechanism of water with silicon-containing materials. First, Doremus established a model for water diffusion and reaction through glassy oxides with one hypothesis concerning the species mobility. For this model, a protective layer is considered to be formed during SiC wet oxidation [81,82] and a two-step mechanism is assumed when water is in contact with the oxide layer.

First, the solution of water molecule is possible by breaking Si-O-Si bridges (30):

H_2_O + Si-O-Si = 2 Si-OH (30)

Then, Doremus assumed that the mobility of dissolved water is higher than that of one of the OH groups which belongs to the network. He suggested that dissolved water molecules jump from one cavity to another without any reaction with Si-O-Si bridges. In silica, the diffusion process proceeds via an interstitial mechanism [83]. However, another mechanism is possible in which water molecules react with Si-O-Si bridges on one site and are regenerated on another site. This is denoted as the interconversion–diffusion model [84].

When Equation (30) reaches equilibrium, the equilibrium constant, $K_{2},\;$is defined by the following equation:

(31) K2=S2C

where S is the concentration of hydroxyl groups and C the concentration of dissolved molecular water. This reaction is bimolecular and this expression assumes that hydroxyl groups can interact with their neighbor to regenerate a water molecule.

The diffusion of water through the oxide scale then needs to be expressed. Fick’s law describes the diffusion of water as a movement of molecules along the *x* axis due to a gradient of concentration. Here, we consider Fick’s second law (32):

(32) ∂Cx, t∂t=D∂2Cx, t∂x2

where x and t represent the distance and time of diffusion and D the water diffusion coefficient (cm^2^/s). The water diffusion coefficient can be calculated empirically through the Arrhénius relation, as diffusion is a thermally activated process (33):

(33) D=D0Tnexp−EART

where $D_{0}$ is the pre-exponential constant, R is the gas constant, $E_{A}$ is the activation energy, T is the temperature and n is a temperature-dependence exponent. Usually, n=0 for simplicity as the activation energy depends on the temperature, so the pre-exponential term has little influence on the diffusion coefficient of water [76].

Secondly, we need to consider the influence of the removal and generation of water molecules on the diffusion of water. Thus, a term (∂Sx, t∂t) is subtracted from Equation (32):

(34) ∂Cx, t∂t=D∂2Cx, t∂x2−∂Sx, t∂t

where S is linked to C by a simple linear dependence in order to solve Equation (33):

(35) K1=SC

This relation assumes that OH groups are immobile which implies that the generation of water molecule is of first order.

Thus, Equation (34) is expressed as follows:

(36) ∂Cx, t∂t=D1+K1∂2Cx, t∂x2

Now, it is clear that the diffusion coefficient of water D has changed and this model defines the effective diffusion coefficient, $D_{e}$, by the following expression (37):

(37) $$D_{e}=\frac{D}{1+K_{1}}$$

where $D_{e}$ is independent of the concentration of molecular water but depends on the initial concentration of hydroxyl group in the network:

➔ When C >> S, the diffusion process is not influenced by the reaction, so $K_{1}\approx 0$ and $D_{e}\approx D$
➔ When S >> C, the effective diffusion coefficient decreases, as the reaction of molecular water occurs in the oxide layer, as described by the equilibrium Equation (31). Therefore, the effective diffusion coefficient takes the following form:

(38) De=2SDK2

where $D_{e}$ becomes dependent of the concentration of molecular water when the generation of water molecule is of second order.

**Figure 22:**
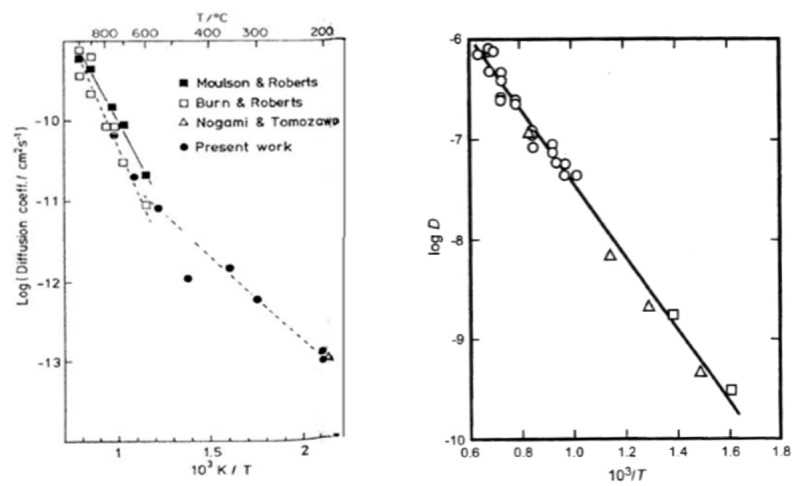
(**Left**) Arrhenius plot of the effective diffusion coefficient of water into silica glass. Reprinted with permission from Reference [85]. Copyright 1989 The American Ceramic Society/Wiley. (**Right**) Arrhenius plot of the diffusion coefficient of water into silica glass. Reprinted with permission from Reference [82]. Copyright 1999 Materials Research Society.

In the literature, some studies reported the temperature and time-dependence of this model, as shown in the left graph on Figure 22.

For temperatures above 600 °C, it was found that the reaction (jump of water) is dominant over its diffusion. Therefore, the concentration of water is low, and hydroxyl groups are the main species. They diffuse in the oxide layer and the reaction is bimolecular. However, below 600 °C, the concentration of exchanged OH groups at the oxide surface is time dependent if the local equilibrium is not reached. Over a short time, OH groups are diffusing in the whole layer due to the presence of defects, and the reaction is bimolecular. However, over a long time, OH groups cannot diffuse far and tend to react with their neighbor, so the reaction is almost unimolecular. For bimolecular reactions, expressions (31) and (38) are applied and for unimolecular reactions, expressions (35) and (37) are valid.

Furthermore, Doremus calculated D, the coefficient of water, from $D_{e}$, the effective coefficient of water, extracted from the data of Wakabayashi and Tomozawa, on the right graph seen in Figure 21 [86,87,88]. Equation (38) is used, and the linear regression shows agreement with the model. The activation energy for water permeation through silica is found to be equal to 70 kJ/mol. Another remark is that the D value is independent of time whereas the $D_{e}\;$value depends on the water solubility, diffusion time, water concentration or pressure, and type of diffusion experiment. However, it should not influence the activation energy.

Finally, Fortier and Giletti were able to correlate $D_{e}$ with the measure of ionic porosity, Z, between 500 and 700 °C and under a water pressure of 100 MPa, calculated by the following [86]:

(39) Z=1−VVc

where V is the total volume of atoms in the unit cell and $V_{c}$, the volume of the unit cell. The ionic porosity represents the openness of the oxide network and shows agreement with the activation energy $E_{A}$ (kJ/mol). It can then be concluded that water is the diffusing species. The linear regression found for water permeation through eighteen glassy and seventeen crystalline oxides is:

(40) $$E_{A}=484-821Z$$

As a result, the higher the activation energy, the lower the ionic porosity, the lower the capacity of the oxide structure to open, and, therefore, the diffusion of water is slower.

To conclude on this model, the diffusion of water and oxygen species through amorphous and crystalline silica proceeds via two routes, according to Lamkin et al. [76]:

− The open porosity of the network allows the permeation of molecular species and,
− The Si-O-Si bridges network provides defects (as lattice vacancies) through which structural self-diffusion occurs with breaking and reforming of the bonds.

For the two diffusion processes, similar activation energies for dry oxidation were determined (with a value of 113 kJ/mol for molecular permeation of oxygen [44] and values between 85 and 121 kJ/mol for oxygen self-diffusion through amorphous scale). Likely, the oxygen transport mechanism is similar in both cases, and this conclusion can be applied for diffusion of water through silica. In fact, activation energies for water permeation are lower than the ones for oxygen, as it is reported in the table below. Doremus showed that this was due to high diffusivity of water in the network which is linked to its high solubility and high reactivity with silanol bonds.

For the parabolic regime, the activation energy is related to the energy needed for oxidant diffusion. As high activation energy was recorded, Doremus stated that, because the diffusion mechanism could proceed via the breaking of Si-O or Si-OH bonds, the diffusion mechanism is defect-based. Contrarily to other authors, he proposed that O_2_ dissociates into atomic oxygen, which implies the diffusion of charged species into the silica scale [82]. According to Narushima et al., O_2_^-^ could be the diffusing species, whereas Singhal hypothesized that hydroxyl ions are the reason for high parabolic activation energy recorded under water vapor. However, for lower values, molecular permeation is the most probable diffusion mechanism [76].

Regarding the linear regime of SiC oxidation, the surface-reaction mechanism is thought to proceed via bond breaking at the SiC/SiO_2_ interface. Some authors proposed two possible pathways: one via the breaking of Si-C bonds, which required an energy of 290 kJ/mol [70]; or the other via the Si-Si bonds breaking, which required an energy of 177 kJ/mol, according to Pauling [73] (see Table 6 above).

### 3.5. Conclusions

The wet (air) oxidation process was expressed, and different parameters were identified as highly influent on the oxidation kinetics of SiC and Si materials. A new model for wet oxidation kinetics is described as the paralinear model, which takes into account the volatilization of the silica scale at high pressures and high gas velocities. Finally, the main conclusions (i.e., effect of impurities and temperature) made for dry oxidation appear to be valid for the wet oxidation. However, the water dramatically increases the oxidation rate of SiC materials, even for small percentages of H_2_O. In fact, water is the primary oxidant in wet oxidation and has the capacity to enhance the oxidation rates by loosening the silica network. As a consequence, impurities and oxygen can diffuse faster in the silica network and enhance the oxidation rates. In fact, the impurities have the capacity to increase the local viscosity of the amorphous layer (i.e., decrease the stress) and limit the growth of cristobalite crystals. In opposition to that, water accelerates the silica devitrification (i.e., the crystallization rate), and the oxidation rates decrease as the layer becomes crystalline. The competition between these two antagonist phenomena could explain the instability of the oxide layer grown during wet oxidation.

Finally, Doremus proposed a diffusion–reaction model to show that diffusion coefficient of water through oxide scales is modified as reaction with the network occurs [80,82]. This model agrees well with the data from the literature, and it shows a time dependence when local equilibrium has not been established. It also shows that diffusion coefficients depend on a variety of factors, but it does not depend on the activation energy of water diffusion. Fortier and Gilletti [86] were able to correlate the ionic porosity with the activation energy of water permeation. A linear relation was obtained, showing that water diffusion increases with the openness of the oxide structure. In this model, water reacts with silanol groups to diffuse until it reaches the SiC interface, which gives rise to hydrothermal oxidation. Therefore, the degradation of SiC materials is caused by water reaction and belongs to the “Hydrothermal Corrosion” classification.

## 4. Hydrothermal Corrosion of Silicon Carbides Materials

**Figure 23:**
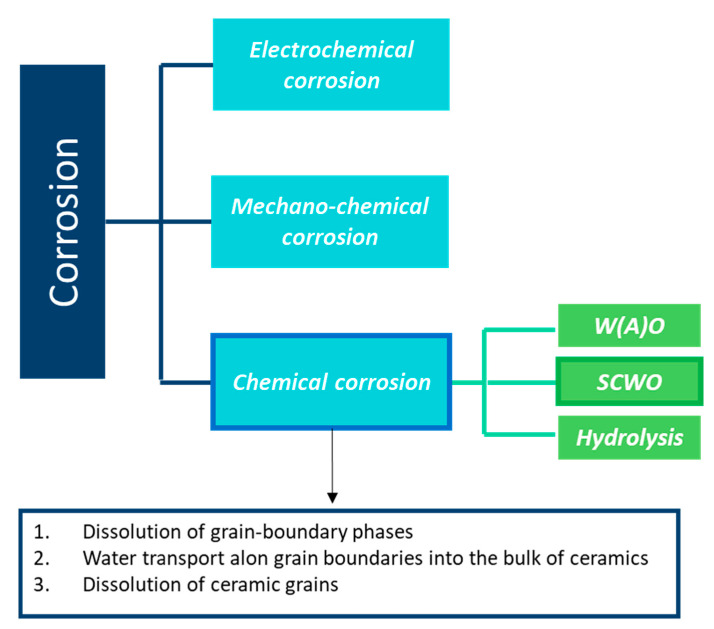
Ordering of the corrosion behavior of ceramic oxide materials. W(A)O stands for wet (air) oxidation and SCWO for supercritical water oxidation.

Under wet atmosphere, the SiC material is highly damaged, and its mechanical properties decrease. Since the hydrothermal oxidation reaction leads to the degradation of the material, it is classified as a chemical corrosion. In Figure 23, the corrosion behavior classification is given.

First, electrochemical corrosion is more developed for metals, as ceramics can be insulators or semiconductors and do not give up electrons easily. Then, mechanochemical corrosion occurs mostly for structural ceramics under thermal or mechanical loading, whereas tribochemical interactions can happen between ceramics and water [90].

Finally, chemical corrosion is the most studied case of corrosion in aqueous environments. In fact, the lack of corrosion resistance in water and water vapor of SiC is due to the formation of stable silicon hydroxides. As the oxides cannot act as a protective barrier, the chemical corrosion leads to rapid consumption of the material by a typical grain dissolution mechanism.

**Figure 24:**
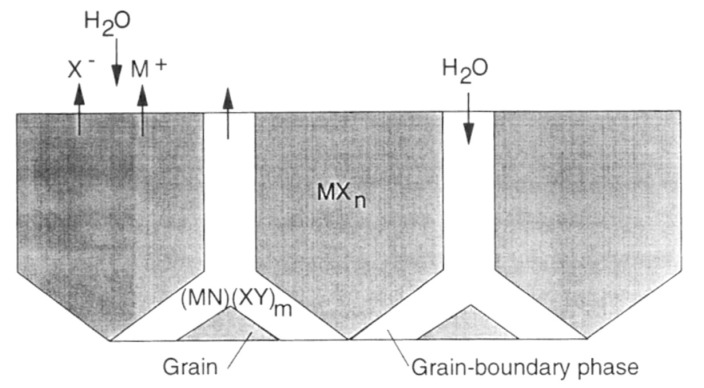
Schematic sketch showing dissolution of grains and grain-boundary phases (mostly silicates) and water transport along grain boundaries into the bulk of ceramics. Reprinted with permission from Reference [90]. Copyright 1994 Materials Research Society.

According to Kim et al., silicon carbides contain grain boundary layers, which drives their corrosion resistance [91]. Thus, the corrosion behavior of ceramics depends not only on composition but on microstructure as well. Under hydrothermal conditions, the chemical corrosion occurs as follows (Figure 24):

1. Reaction of grain-boundary phases,
2. Water transport along grain boundaries into the bulk of ceramics,
3. Reaction of ceramic grains.

In order to better understand this corrosion mechanism, several reaction models were proposed to explain the SiC/water interaction.

### 4.1. Reaction Model for Chemical Corrosion

#### 4.1.1. Yoshimura’s Model for Hydrothermal Oxidation of SiC in Supercritical Water

From previous studies [35,36,37], the hydrothermal oxidation of SiC was thought to release CO_2_ species, as follows (41):

SiC + 4H_2_O → SiO_2_ + CO_2_ + 4H_2_ (41)

However, Yoshimura et al. discovered the formation of a large amount of CH_4_ in comparison to CO_2_ [92] and concluded that the oxidation reaction was (42) [58]:

SiC + 2H_2_O → SiO_2_ + CH_4_ (42)

In this model, no reactions occurred below 500 °C even at 100 MPa H_2_O, whereas above, a weight gain was observed. Thus, SiC material is transformed into amorphous silica, which crystallizes to form cristobalite and tridymite above 700 °C.

**Figure 25:**
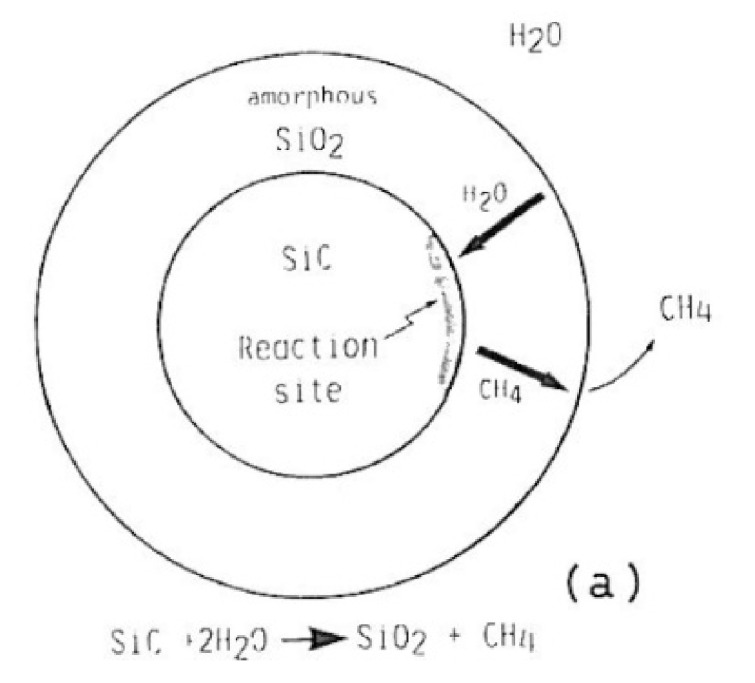
Reaction model of the hydrothermal oxidation of SiC powder. Reprinted with permission from Reference [92]. Copyright 1986 Materials Research Society.

The following reaction model is proposed for SiC oxidation in H_2_O, as it is represented on Figure 25 below. After the amorphous silica layer is formed on the surface of SiC particles, H_2_O and CH_4_ diffuse, respectively, inward and outward.

It is probable that non-polar species encounter difficulties to diffuse through the amorphous silica. Thus, the oxidation rate of SiC in H_2_O seems to be controlled by the outward diffusion of CH_4_, or by the separate diffusion of carbon and hydrogen, if CH_4_ has dissociated in the amorphous silica. The hydrothermal oxidation of SiC could proceed by two simultaneous reactions (43) and (44):

SiC + 2H_2_O → SiO_2_ + CH_4_ (43)

CH_4_ + 2O_2_ → CO_2_ + 2H_2_O (44)

At a high temperature, reaction (22) is more stable than the reaction (45), and water could oxidize H_2_ (46):

SiC + 4H_2_O → SiO_2_ + CO_2_ + 4H_2_ (45)

4H_2_ + 2O_2_ → 4H_2_O (46)

Thus, the oxidation of SiC in hydrothermal medium proceeds via a two-step mechanism instead of a one-step reaction (47):

SiC + 2O_2_ → SiO_2_ + CO_2_ (47)

Yoshimura et al. noticed that the activation energy under 10 MPa (194 kJ/mol) was higher than the one calculated at 100 MPa (167 kJ/mol). Indeed, the oxidation rates were accelerated when high water pressure is used, and these two values were slightly smaller than for dry oxidation (above 200 kJ/mol). Therefore, it was concluded that, firstly, the water pressure did not affect the oxidation mechanism and, secondly, that the diffusing species might differ under dry oxygen and pressurized water.

The reason for this is that H_2_O diffuses faster than oxygen in the silica layer, so the kinetics of reactions (43) plus (44) or (45) plus (46) would be higher than for the reaction (47). This could be the key element for higher oxidation of SiC under hydrothermal conditions. However, Hirayama et al. proposed that it might be due to the formation of hydrosoluble silica scale.

#### 4.1.2. Hirayama’s Model for SiC Corrosion in Water Vapor

**Figure 26:**
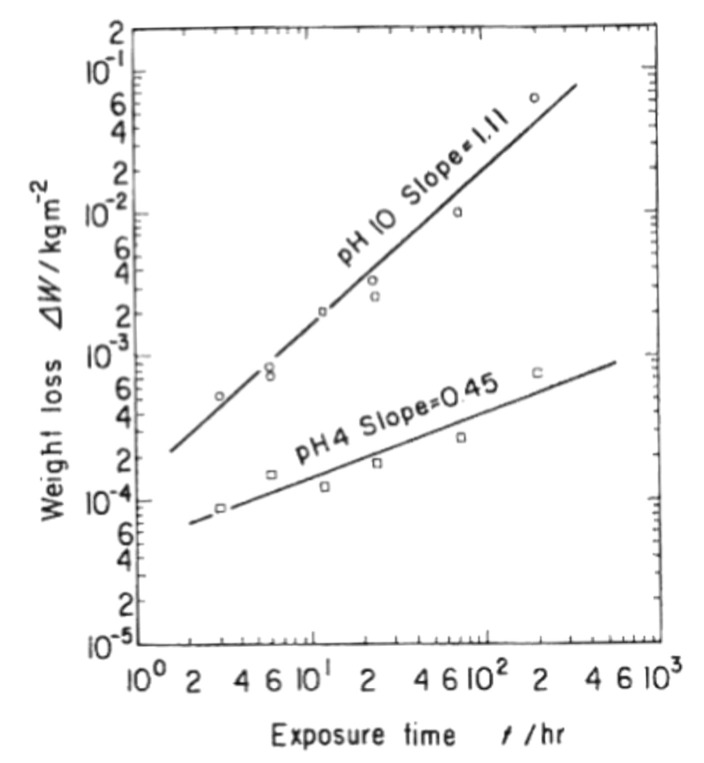
Weight change–time diagram for SiC ceramics immersed in an oxygenated solution (dissolved [O_2_] is 32 ppm) at 290 °C. Reprinted with permission from Reference [93]. Copyright 1989 The American Ceramic Society/Wiley.

In 1989, Hirayama et al. [93] investigated the corrosion behavior of silicon carbide ceramics after immersion in 290 °C water solutions with different pH. After a 72-h exposure, the exposed α-SiC materials demonstrate a higher dependency on pH for weight loss in oxygenated water than in deoxygenated water. Figure 26 relates their results:

From the model of SiC dissolution presented by Yoshimura et al., the following reaction is expected to occur [92]:

SiC + 2H_2_O → SiO_2_ + CH_4_ (48)

This reaction contributes to the weight gain because solid SiC changes into solid SiO_2_. However, Hirayama et al. found out that the weight of the materials decreased with the exposure time, as silica easily dissolves in alkaline water, following the below-mentioned reaction (46):

SiO_2_ + H_2_O = H_2_SiO_3_ = HSiO_3_^−^ + H+ = SIO_3_^2−^ + 2H^+^ (49)

Thus, two protons are generated, so the reaction is pH-dependent. The higher the pH values, the more the reaction can be accelerated, and the larger the weight loss. Indeed, a high dissolution rate of SiO_2_ films is recorded for high pH values. Moreover, when oxygen is dissolved in the solution, reactions (50), (51 and (52) seem to participate in the SiC oxidation:

SiC + 2O_2_ → SiO_2_ + CO_2_ (50)

CH_4_ + 2O_2_ → CO_2_ + 2H_2_O (51)

CH_4_ + 2O_2_ → 2H^+^ + CO_3_^2−^ + H_2_O (52)

The reaction (50) and (48) contribute to weight gain. The reaction (52) can shift the equilibrium of reaction (48) to the right and then contributes to weight gain rather than losses. However, the authors are not able to explain the important weight loss generated by oxygenated water in comparison with deoxygenated water. As SiO_2_ is not identified on the surface (SEM, X-ray or AES), no production of SiO_2_ is assumed, and another dissolution model is proposed, in which a poorly adherent layer, such as Si (OH)_4_, is produced on the surface:

SiC + 4H2O → Si(OH)4 + CH4 (53)

with the dissolution reaction (53) as follows:

Si(OH)_4_ → H_3_SiO_4_^−^ + H^+^ → H_2_SiO_4_^2−^ + 2H^+^ (54)

In the case of oxygenated solution, the oxidation of SiC is as follows:

SiC + 2O_2_ + 2H_2_O → Si(OH)_4_ + CO_2_ (55)

Again, the CO_2_ produced dissolves in water as follows:

Si(OH)_4_ + CO_2_ + H_2_O → H_3_SiO_4_^−^ + HCO_3_^−^ + 2H^+^ → H_3_SiO_4_^−^ + CO_3_^2−^ + 4H (56)

This model demonstrates that reaction (53) is linked to the equilibrium of reaction (54) in deaerated solution, and the reaction (56) dominates the equilibrium of reaction (55), in aerated solution. Therefore, the reactions (54) and (56) contribute to weight loss because of the dissolution of the substrate. As the pH increases, the equilibrium of reactions (53) and (55) shifts to the right, so more proton ions (H^+^) are released. In the case of oxygenated water, the oxidation reaction (56) produces four H^+^, whereas for deoxygenated water, only two are released by the reaction (54). This results in higher weight loss for oxygenated solution.

**Figure 27:**
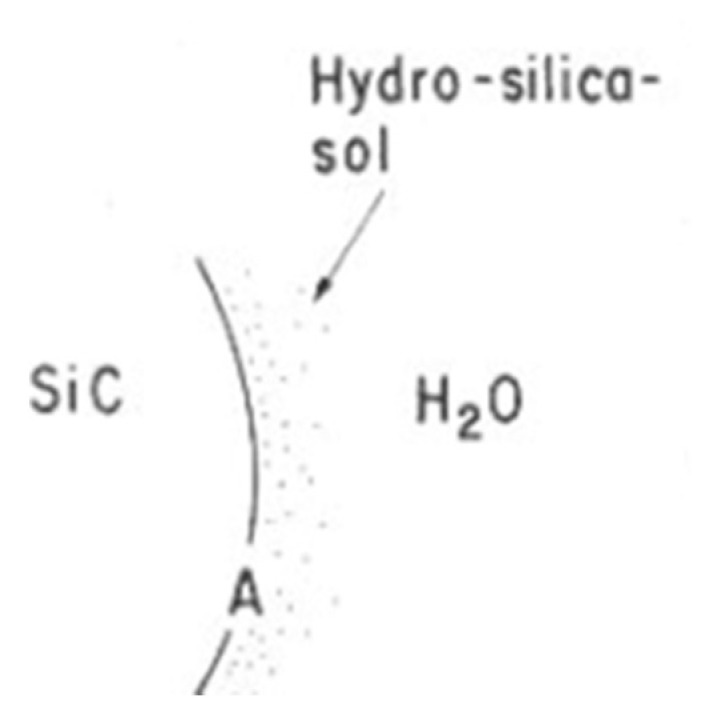
Dissolution model, assuming that a hydro–silica–sol is produced. Reprinted with permission from Reference [93]. Copyright 1989 The American Ceramic Society/Wiley.

The new model is illustrated in Figure 27:

The overall hydrothermal oxidation reactions with SiC materials in alkaline medium are as follows:

− For a deoxygenated solution:
  SiC + 4H_2_O = H_2_SiO_4_^2−^ + 2H^+^ + CH_4_ (57)
− For an oxygenated solution:
  SiC + 2O_2_ + 2H_2_O = H_3_SiO_4_^−^ + CO_3_^2−^ + 4H^+^ (58)

This model shows that the hydrothermal oxidation of SiC materials is not able to produce a protective silica layer to prevent further oxidation. Furthermore, multiple oxidation reactions occur with oxygen as the primary oxidant species.

The conclusion is that the production of a non-protective hydrosoluble silica layer is the key element which allows for the understanding of the high oxidation rate of SiC in water. Furthermore, the multiple roles of oxygen are proof of high synergy between water and oxygen, which leads to higher oxidation rates. Finally, as these hydrothermal reactions are pH-dependent, they are not considered as oxidation reactions but instead related to hydrolysis. This point is discussed in the following section on Allongue’s model.

#### 4.1.3. Allongue’s Model for Dissolution of Silicon in Liquid Water

An in situ scanning tunneling microscopy (STM) is used to investigate the etching of Si (111) in alkaline solutions [94]. During the dissolution process, the silicon surface is covered with Si-H bonds followed by the formation of etch pits. The etch rates are deduced from the weight loss of the material and a model of chemical dissolution of Si-crystals is designed [95].

**Figure 28:**
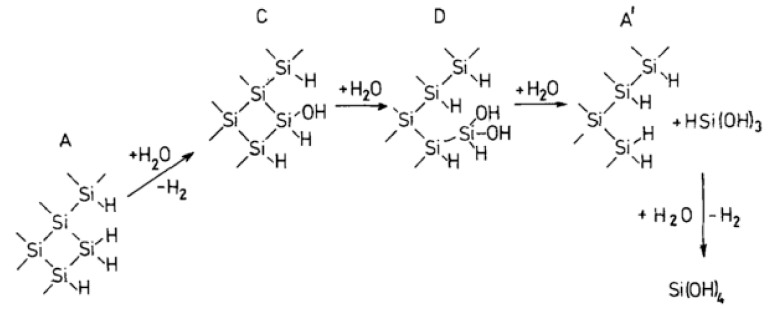
Main reaction path for the chemical dissolution of Si. Reprinted with permission from Reference [94] Copyright 1993 IOP.

In this model (Figure 28), the dissolution occurs at a kink site, (structure A on Figure 28). A kink site is a defect in the crystal where one Si atom cannot be involved within four bonds with other Si atoms. As this site is located at the edge, the kink Si atom has only two bonds linked to the crystal lattice while the two other bonds are involved in Si-H bonds. The structure A evolves into C, and then D and A’ by successive hydrolysis. At the end, the last product is decomposed to give Si(OH)_4_.

These successive steps are described in detail:

First, the direct Si-Si backbones hydrolysis occurs (59):

Si-Si + H_2_O → Si-H + Si-OH (59)

The first step of Si-H bonds hydrolysis released H_2_ as follows (60):

Si-H + H_2_O → Si-OH + H_2_ (60)

The third step of hydrolysis produces Si(OH)_2_ (61):

Si-OH + H_2_O → Si(OH)_2_ (61)

Then, the secondary product HSi(OH)_3_ is obtained (62):

Si(OH)_2_ + H_2_O → HSi(OH)_3_ (62)

A second H_2_ molecule is produced by the decomposition of the primary product HSi(OH)_3_ in solution (63):

HSi(OH)_3_ + H_2_O → Si(OH)_4_ + H_2_ (63)

The final product entering the solution is silicic acid, Si(OH)_4_, or its equivalent dissolution product in alkaline solution (64):

Si(OH)_4_ + H_2_O → [SiO_4_H_2_]^2−^ (64)

So, the overall reaction produces the silicate oxyanions and releases hydrogen (62):

Si + 2HO− + 2H_2_O → [SiO_4_H_2_]^2−^ + 2H_2_ (65)

Regarding the kinetics of dissolution, the limiting step could be the A→C or A→D chemical paths.

**Figure 29:**
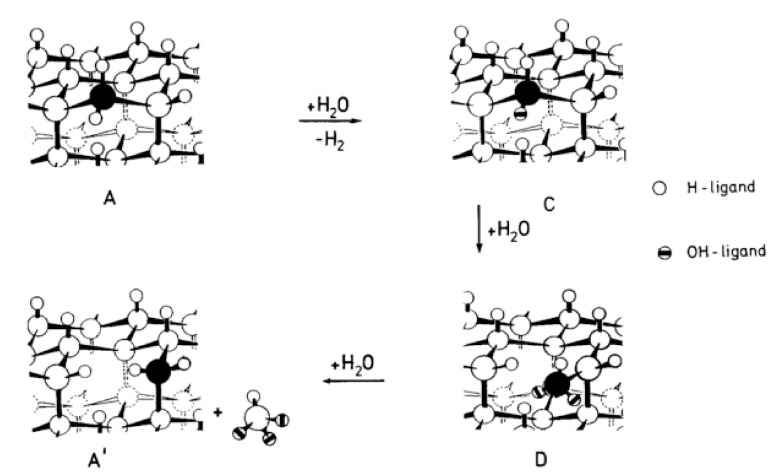
Three-dimensional model for the chemical dissolution of a kink Si atom (black), from the structure A to A’. Reprinted with permission from Reference [95]. Copyright 1993 IOP.

The key element for all these reactions to occur is that the surface is exhibiting H terminations (see Figure 29). Therefore, a positive charge is induced by the polarization of the Si bond with OH or H ligands. This leads to the attachment of the OH ligand on the kink atom site, and the H ligand on the Si atom is underneath when the Si-Si backbone is hydrolyzed.

During the consecutive hydrolysis steps of silicon, the addition of OH ligand induces the polarization of the Si-OH bond, which dislocates the kink site atoms. Due to this, is the O-H ligand quickly removed from the lattice.

Finally, the OH^-^ ions catalyze the hydrolysis of Si-Si and Si-H bonds (59) and (60) and allow for the dissolution of hydrolysis products. Hydroxyl ions then tend to enhance the hydrolysis of Si.

This model demonstrates good agreement with the Hirayama’s model of SiC dissolution. Indeed, if silicon is substituted by SiC in Equation (65), the hydrolysis reactions will lead to the release of methane gas instead of hydrogen (66):

SiC + 2HO^−^ + 2H_2_O → [SiO_4_H_2_]^2−^ + CH_4_ (66)

If only water molecules participate to the hydrolysis, Equation (67) is obtained:

SiC + 4H_2_O = [SiO_4_H_2_]^2−^ + 2H^+^ + CH_4_ (67)

Now, one can see that this equation is equivalent to the hydrothermal oxidation expressed by Hirayama’s model for deoxygenated solutions. Therefore, its validity is confirmed.

#### 4.1.4. Conclusions

According to the Yoshimura’s model, a silica scale is formed during SiC corrosion through which oxidant species and volatile by-products diffuse. He concluded that water diffused faster than oxygen due its diffusion reaction into oxide scales, according to the Doremus model.

In opposition to that, Hirayama’s model does not consider the formation of a silica scale on the SiC surface. Several reactions were expressed for which hydrosoluble silica is produced but neither protective nor non-protective silica were identified.

Allongue’s model suits Hirayama’s model well. Indeed, water is found to hydrolyze silicon by a five-step mechanism with the production of silicic acid. Still, what can be considered about a hydrolysis mechanism that is valid for SiC interface?

Indeed, a microstructure study can reveal if the corrosion mechanism is the same for silicon and silicon-containing materials. In the next section, the corroded surface of such materials is compared.

### 4.2. Hydrothermal Corrosion of the Surface

Allongue et al. conducted in situ STM etching of Si (111) face in alkaline solution [94] and reported the etching mechanism of the Si double layer steps. In fact, the Si (111) face consists of smooth terraces separated by a single double layer of 3.14 Å height, at the edge of which kink sites are found.

The first observation is that silicon can be hydrolyzed by water and this reaction is catalyzed by hydroxyl ions. At high pH values, the hydrolysis becomes dominant over the electrochemical mechanism responsible for the Si corrosion.

**Figure 30:**
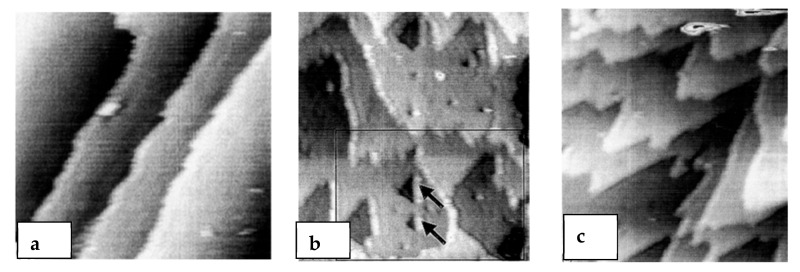
In situ STM observation of the surface of n-Si(111) in 2 M NaOH solution under relatively strong hydrogen evolution of 200 µA/cm^2^ (**a**) Image (380 × 380 Å^2^) of the starting material. The following tunneling conditions: U_S_ = −0.63 V/Pd-H; I_S_ = −150 µa/cm^2^; tip: U_T_ = +300 mV/Pd; IT = 0.2 nA (**b**) Image (1076 × 1193 Å^2^) of the growth of triangular etch pits which is followed by their coalescence. The following tunneling conditions: U_S_ = −0.63 V/Pd-H; I_S_ = −150 µa/cm^2^; tip: UT = +300 mV/Pd; IT = 0.2 nA (**c**) Image (1280 × 1470 Å^2^) at the end of corrosion under the same tunneling conditions as (**b**) and terraces go upwards from the bottom left to the upper right. Reprinted with permission from Reference [94]. Copyright 1992 Elsevier.

The second observation is that at negative bias, the Si etching proceeds by shrinkage of the terraces and an increase of the surface roughness. This so-called “step-flow” mechanism competes with the nucleation of etch pits at cathodic bias. The corrosion evolution of the Si surfaces is reported on the Figures below, as in Figure 30. The picture on the left side shows the smooth terraces of the starting Si materials. As the corrosion occurs, pit formation and growth are observed on the terraces, as it is seen in the center image. After the coalescence of the etch pits, the corroded surface reveals sharp edges. The surface topography on the rightmost image is consistent with corrosion etching via a step-flow mechanism and etch pit formation.

Corrosion pitting is also highly dependent on the chemical environment, along with the homogeneity and morphology of materials. Smialek et al. showed that the hot corrosion of α-SiC proceeded via preferential attack at structural discontinuities, whereas excessive corrosion of pressures sintered β-SiC produced bubbles and resulted in high-roughness surfaces due to high carbon content [96]. Indeed, Henager et al. claimed that the preferential formation of bubbles or localized corrosion along grain boundaries or aligned with grains is due to higher dissolution rates at boundaries [97].

After exposure of CVD-SiC materials to deoxygenated water at 300 °C and 10 MPa, the surfaces revealed both embryonic and large pits, after initial exposure for 4000 h. The small pits could have been produced by the formation of volatile carbon species, such as CO, CO_2_ or CH_4_ that act to disrupt the surface silica layer. Locally, the CO and CO_2_ would increase the acidity and accelerate the corrosion process, resulting in the formation of local pits due to the agglomeration of small pits on the surface.

Therefore, pitting degradation affects the mechanical properties of SiC materials, and there is a need to identify which combustion conditions lead to the highest strength reductions.

Hirayama et al. carried out the first microstructural studies of SiC materials revealing that corrosion occurred at grain boundaries and suggested a preferential intergranular attack [93]. Kim et al. confirmed this observation by performing corrosion experiments on sintered SiC (SSiC) and chemically deposited SiC (CVD-SiC) ceramics, in distilled water at 360 °C and over 10 days [90]. As the CVD-SiC has higher purity than SSiC and clean grain boundaries, the sample showed a stronger corrosion resistance.

**Figure 31:**
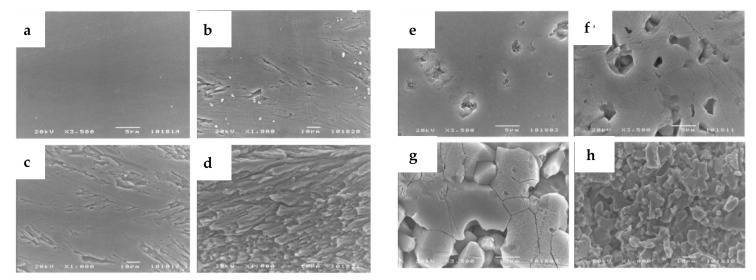
Surface microstructures of the (**Left**)—(**a**) as-polished and corroded SSiC specimens after corrosion testing for (**b**) 1, (**c**) 5 and (**d**) 7 days in water at 360 °C and (**Right**)—(**e**) as-polished and corroded CVD SiC specimens after corrosion testing for (**f**) 3, (**g**) 7 and (**h**) 10 days in water at 360 °C. Reprinted with permission from Reference [91]. Copyright 2003 Springer Nature.

In fact, in SiC samples, boron and carbon atoms are known to be segregated at grain boundaries, and during corrosion, these impurities are rapidly attacked. As a result, the disintegration of grains into water occurred due to the weakening of grain boundaries at extended corrosion times. On the contrary, the CVD-SiC specimen underwent low weight loss and the corroded surface showed feature like the columnar growth pattern of the SiC deposits, it can be seen on Figure 31, below:

No SiO_2_ or Si(OH)_4_ amorphous layer was observed because of the very thin thickness of the film or due to its instability. According to Hirayama, Kim et al. suggested that the oxidation of SiC material produces a poorly adherent film: Si(OH)_4_ instead of SiO_2_, in high-temperature water. However, Barringer et al. [98] recorded lower linear mass loss for CVD-SiC exposure to deoxygenated water at 360 °C and assumed that the control of oxygen helped to reduce corrosion rates. Finally, the corrosion behavior of reaction-bonded silicon carbide (RBSC) in pure water at 360 °C showed an increase of the weight loss with the amount of free Si atoms in the material [99].

**Figure 32:**
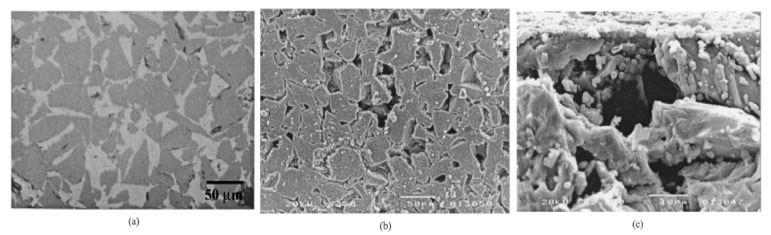
Optical micrograph of the RBSC specimen before corrosion test (**a**) and SEM micrographs of the surface (**b**) and the cross-section (**c**) of the same specimen after corrosion at 360 °C for 7 days. Reprinted with permission from Reference [99]. Copyright 2002 Springer Nature.

In Figure 32 below the light and dark gray phases represent the Si phase and SiC particles, respectively. During corrosion, the Si-free phase was preferentially corroded in high-temperature water, which led to the formation of large voids in the material.

Recently, Park et al. [100] carried out long-term corrosion tests on high-purity CVD-SiC specimens in water and steam up to 400 °C. They showed that the dissolved oxygen content was the most dominant factor controlling the corrosion of SiC in water. The grain boundary dissolves preferentially during the early stage of corrosion. The grains detach from the surface when the grain boundaries become thinner, thereby leading to an acceleration of the weight loss.

**Figure 33:**
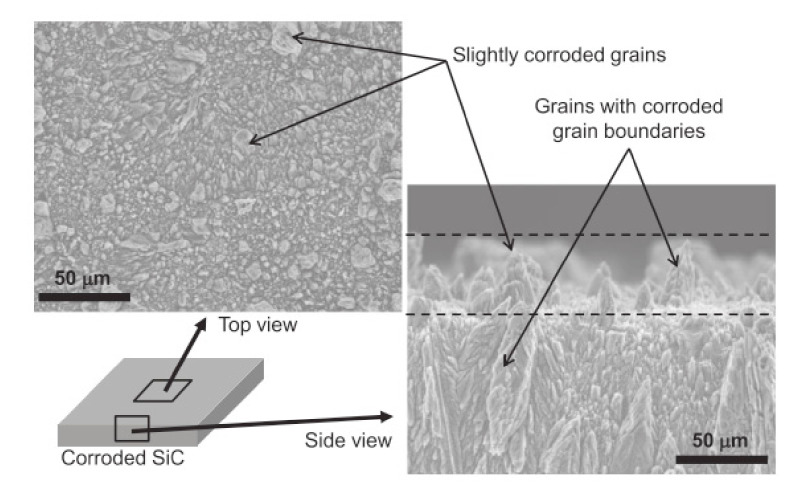
Scanning electron micrographs of the surface morphology of the CVD Si corroded in the 360 °C static water autoclave for 90 days. Reprinted with permission from Reference [100]. Copyright 2013 Elsevier.

**Figure 34:**
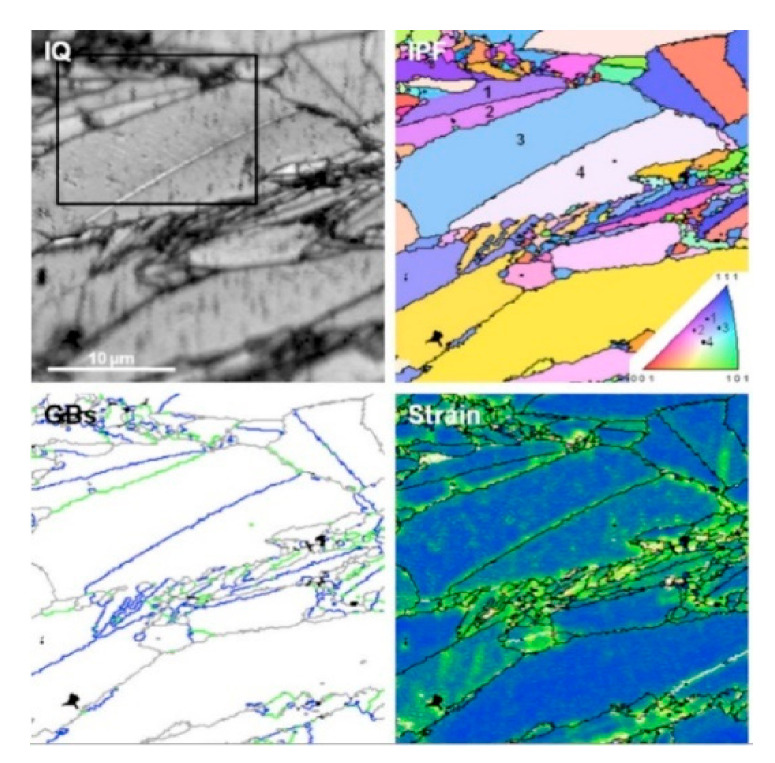
Electron Back-Scatter Diffraction (EBSD) analysis of the cross-section surface (28 × 28 μm) of the sample exposed to the SCW with 10 ppb oxygen for 333 h. GBs denote grain boundaries with blue, green and gray lines denoting three different network, and general boundaries. Reprinted with permission from Reference [101]. Copyright 2009 Elsevier.

In Figure 33, above, the top view shows significant dissolution of the SiC throughout the surface. In the side view, the shapes of many grains are exposed because of the preferential dissolution at the grain boundary during the corrosion process. These observations were confirmed by Tan et al. [101] who exposed CVD-SiC materials to 500 °C water under 25 MPa (Figure 34). The image of strain distribution and Image Quality revealed that corrosion primarily occurred at regions with high intensity of strains associated with small grains.

It was concluded that the pre-oxide was not able to protect the SiC material from dissolving into the water, but it helped to mitigate the dissolution of the SiC.

To conclude on the microstructure study of SiC corrosion, Barringer et al. [96] believed that corrosion occurs via hydrolysis to hydrated silica species at the surface, which are then rapidly dissolved into the water. This conclusion is based on no analytical methods revealing the presence of a measurable oxide scale. In addition to that, a reduction of the amount of oxygen present at the surface was recorded as the exposure time increased. This may be consistent with the removal of Si atoms to form Si(OH)_4_ via water hydrolysis, which allows the Allongue’s model to be extended to SiC materials. Finally, Henager et al. showed that pits resulted from the formation of a local galvanic cell, which was due to hydrolysis reactions involving the dissolution of silica and the formation of carbon [97]. However, tribocorrosion process can lead to such observations—the next section details the tribochemical corrosion of SiC surfaces.

### 4.3. Reaction Model for Tribochemical Corrosion

Under dynamic conditions, the exposure of non-oxide ceramics to oxidizing atmosphere leads to the formation of an oxide film and it is referenced as “tribo-oxidation” whereas the formation of a chemical layer under hydrothermal conditions is referenced as “tribochemistry”. Tribo-oxidation leads to the modification of SiC physical properties, and, according to the wear oxidation model of Quinn, tribo-oxidation is a consequence of a local temperature rise when the sample undergoes friction [102,103].

Both action of friction and temperature (because of fast kinetics) influence the tribochemical SiC degradation. Some studies reveal that the higher the humidity, the lower the coefficient of friction and wear rates [104,105] and Zum et al. demonstrated that when SiC is submitted to water, tribochemical polishing of its surface is expected due to boundary lubrication [106].

Therefore, water seems to affect the tribochemical behavior of SiC. However, the effect of water, including its temperature and pressure, is not clear. Hence, Presser et al. studied the hydrothermal behavior of SiC materials and its wear properties evolution [107] by conducting degradation experiments under static (at 500 °C up to 700 MPa) and dynamic (tribological) conditions in water. They showed that the smoothing process of SiC was responsible for the decrease of the surface roughness and the increase of the sliding behavior, which resulted in the decrease of the friction coefficient. As well, silicon leaching and amorphization of the surface occurs under tribological tests in water medium (without pressure).

**Figure 35:**
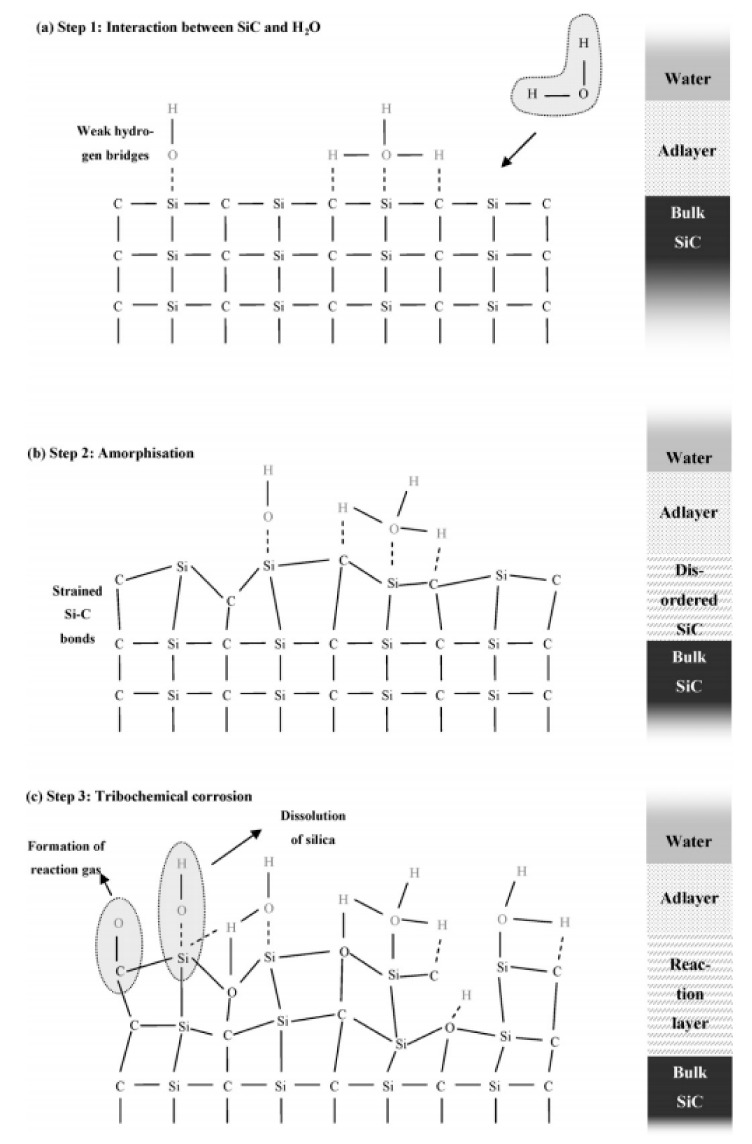
Three-step tribomodel of SiC corrosion. Reprinted with permission from Reference [107]. Copyright 2009 Elsevier.

However, the hydrothermal degradation leads to pitting formation and an increase of the roughness with bubbles and silica precipitates which agrees with the observations of Barringer et al. [98]. Finally, a tribochemical wear model is designed into three steps (see Figure 35):

1. Interaction between SiC and water: The bulk reaction leads to the formation of OH groups and to the saturation of dangling bonds. Moreover, weak hydrogen bridges are created.
2. Amorphization: Initially, mechanical stress causes the superficial amorphization of SiC. Therefore, disordered layers and strained Si-C bonds might form with higher susceptibility to be attacked by water. However, neither silica nor oxycarbidic phases were identified knowing that the detection limit of XRD and Raman spectroscopy is about 100 nm.
3. Tribochemical corrosion: Simultaneously, silica dissolves in water, and, for low water-to-SiC ratios, it precipitates. Likewise, a cavitation-like wear phenomenon created by the release of gaseous compounds can cause the delamination of the layer.

In conclusion, this is another corrosion mechanism can lead to the degradation of SiC materials under hydrothermal conditions. This corrosion, tribocorrosion, is enhanced by water, as the dissolution of amorphous SiC is possible by the formation of hydrosoluble silica and by the release of volatile carbonaceous species. Simultaneous hydrothermal corrosion also occurs at grain boundaries.

### 4.4. Conclusions

From the microstructural studies, the etching of Si materials seems similar to that of SiC materials. Indeed, the degradation of silicon-containing materials occurs via pit formation and growth on the surface, which results in high weight losses. The corroded surface shows a high degree of pitting corrosion via preferential attack at the grain boundary, which highly depends on the chemical environment and the morphology of SiC samples. For CVD-SiC, typical columnar corrosion shape is observed, and for SSiC, the etching of carbon and boron atoms occurs locally on its surface. Finally, the degradation process depends on the sample purity: CVD-SiC samples show high corrosion resistance, whereas the presence of Si-free atoms in the RBSC sample leads to a high degradation rate. Moreover, the etching of Si-free atoms is thermodynamically favored, as the corrosion products are stable (silica easily dissolves in water); however, several authors were not able to detect the dissolved silica [98,99,100]. Nevertheless, these authors agreed with the Hirayama’s model of SiC dissolution by water [93] but not with the one of Yoshimura which suggests the formation of a silica scale. These findings also agree with the Allongue’s model for the dissolution of silicon crystals. Indeed, it appears that successive hydrolysis of the Si-Si, or Si-C bonds in the case of SiC materials, causes the formation of OH groups and leads to the etching of Si atoms.

In conclusion, the corrosion behavior of SiC materials seems complex, as several chemical reactions compete, and different mechanisms are taking part in the degradation of the materials, as is suggested by the tribochemical corrosion model.

In the final part of the review paper, the water corrosion of these materials under sub- and supercritical conditions is examined. Several chemical reactions are in competition depending on the temperature and pressure conditions along with the molar ratio of H_2_O:SiC. Indeed, hydrothermal oxidation leads to a silica layer formation whereas hydrolysis reaction leads to a carbon film formation on top of SiC substrates (powders, fibers and substrates).

## 5. Supercritical Water Corrosion of Silicon Carbide Materials

### 5.1. Supercritical Water Characteristics

**Figure 36:**
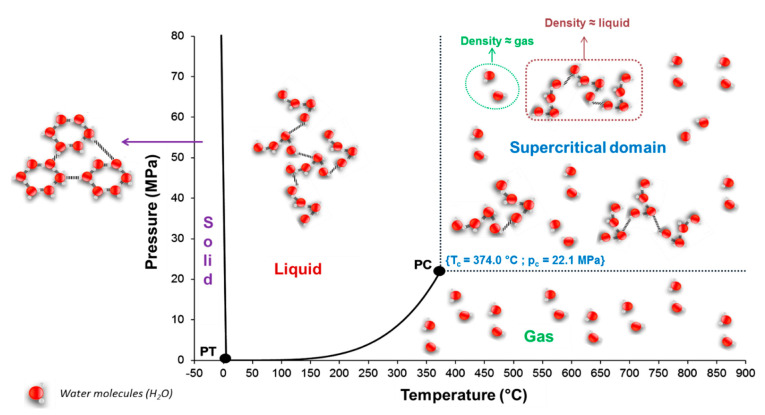
Pressure–temperature phase diagram of pure water. TP is the triple point (T_TP_ = 0.01 °C, P_PT_ = 612 Pa) and CP is the critical point (T_CP_ = 374.1 °C, P_CP_ = 22.1 MPa). Water molecules are draw schematically (planar view) for each state—solid, liquid and gas—and in the case of the supercritical conditions. Reprinted with permission from Reference [108]. Copyright 2010 Wiley.

High temperature and high-pressure medium needs to be defined to have an insight of the interesting properties that a fluid can demonstrate under these particular conditions. Critical coordinates are defined for all pure components and represent the beginning of the supercritical domain. By increasing the temperature and the pressure, the equilibrium state of water evolves as it is shown on the diagram below:

As seen on Figure 36, the three states of water are in equilibrium at the triple point defined for T_TP_ = 0.01 °C and P_PT_ = 612 Pa.

At high temperature and high pressure, the liquid-vapor equilibrium curve ends when the supercritical point of water is reached (T_CP_ = 374 °C and P_CP_ = 22.1 MPa). From there, the matter is called fluid as it demonstrates physicochemical properties in between those of liquid and gas phases.

Indeed, the main characteristics of supercritical fluids (SCF) are low viscosity and no surface tension, which gives rise to high diffusion properties. The SCF region shows discontinuities, as some micro-domains demonstrating liquid-like properties coexist with others which exhibit gas-like properties. Finally, the fluid density evolves linearly with the increase of temperature and pressure.

**Figure 37:**
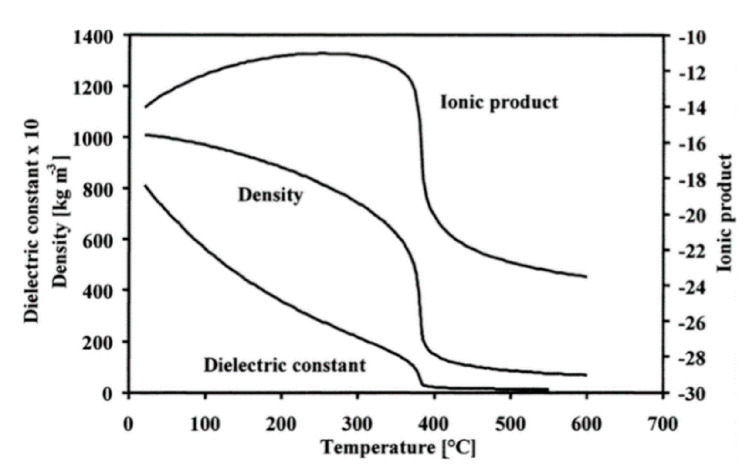
Course of ionic product, density, and dielectric constant of water versus temperature at a pressure of 24 MPa. Reprinted with permission from Reference [109]. Copyright 1999 Elsevier.

Under high pressure and high temperature conditions, the dielectric constant and the density of water sharply decrease. This can be seen with the increase of temperature at a constant pressure of 24 MPa in Figure 37. However, the ionic product reaches a maximum at 300 °C before falling, similar to other parameters.

In other terms, salt dissolution ability of water decreases with the density due to the increase of temperature. The distance between water molecules increases, which weakens the hydrogen bonds and dipole electrostatic interactions. Therefore, low-density water acts as a non-polar solvent, in which salts precipitate, in the supercritical region [110]. However, for a constant temperature of 400 °C, the density, ionic and dielectric constants increase with the pressure. High-density water demonstrates high solvency for inorganic compounds but keeps its solvent-like properties [108]. These observations evidence the high tunability of water properties by playing with the temperature and/or pressure parameters.

Table 7 highlights the existence of another region called the sub- or near critical region which defines water in the liquid state for a temperature range of 250 °C < T < 450 °C and P < Pc [111]. 

**Figure 38:**
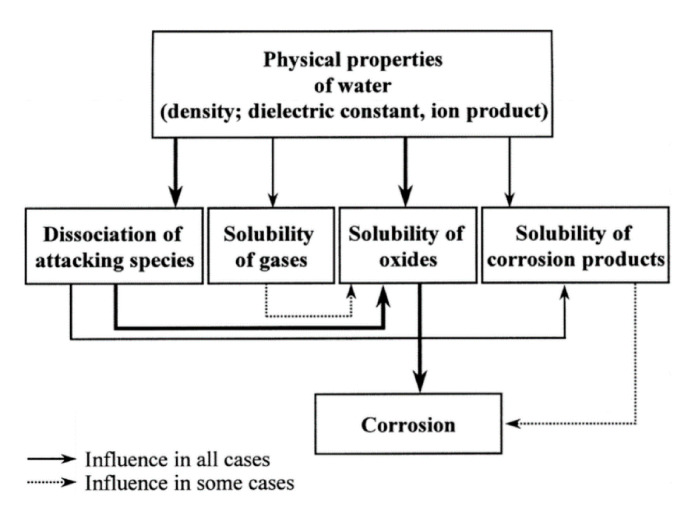
Interdependences of the corrosion-determining factors in high-temperature aqueous solutions. Reprinted with permission from Reference [109]. Copyright 1999 Elsevier.

In the subcritical region, water has infinite compressibility, and, near 250–300 °C, the ionic constant reaches its maximum. Consequently, water acts as an acid or base catalyst and ionic reactions are favored over radical ones. Thus, the electrochemical corrosion is enhanced. Moreover, high-density water and high temperature allows fast reaction kinetics which makes the sub- and supercritical region a high corrosive medium. Kritzer illustrated the corrosion-determining factors and their interdependences below (Figure 38) [109]:

The most used supercritical fluids are water, alcohol and carbon dioxide but the interest for supercritical ammonia and halogenated gases is increasing [112]. Supercritical fluids fill applications to address environmental challenges for wastewater treatment [113] chemical recycling [114] and extraction process [115]. SCFs are involved in organic reactions, for example, in catalyst [116,117], and polymer processing [118]. Finally, SCFs take part in material engineering for synthesis [119,120], surface treatment [121], microfluidics [122] and recently, in nanotechnology [123] and nanomedicine [124].

To conclude, the chemical versatility of sub- and supercritical medium is nearly infinite, as the water properties can be tuned by varying the temperature and pressure conditions. Hence, water can demonstrate high solvent power for organic, salts and gases, along with varying properties from liquid-like to gas-like. This section aims to highlight the different roles of water and its interaction with the matter when using high temperature and high-pressure conditions. More particularly, the interaction between silicon carbide and high pressure/high temperature water is discussed.

### 5.2. Reaction Model of the SiC Hydrothermal Oxidation

The work of Gogotsi and Yoshimura consists of the application of hydrothermal technology to SiC fibers from the studies of Hirano et al. [125] and Yoshimura et al. [92].

Hirano shown that the hydrothermal treatment of SiC at 900 °C and 1000 MPa water pressure enhanced graphitization of carbonaceous materials by the formation of intermediates and gaseous compounds.

Yoshimura et al. studied the interaction of SiC materials with H_2_O under hydrothermal conditions between 400 and 800 °C, at pressures up to 100 MPa and for 72 h and found that the effects of water vapor on the oxidation of SiC powders was of interest, but no model was hypothesized on the reaction of H_2_O and SiC [92].

Surprisingly, Gogotsi and Yoshimura demonstrated that the corrosion of SiC materials (α-SiC single crystals, α-SiC platelets, α and β-SiC powders and β-SiC whiskers) leads to the formation of carbon film above 500 °C through the following relation (68) [126,127]:

SiC + 2H_2_O → SiO_2_ + C + 2H_2_ (68)

Gogotsi and Yoshimura worked on the oxidation and hydrothermal corrosion of SiC by first investigating the hydrothermal corrosion of SiC-based fibers at high temperature and high-pressure water [127]. The experiments were performed on Tyranno fibers under 100 MPa in distilled water at 300–600 °C for 25 h. It was observed that above 300–450 °C, a smooth and uniform carbon film was grown via the following reaction (69):

SiC_x_O_y_ + nH_2_O → SiO_2_ + xC + nH_2_ (69)

In this reaction, the free carbon is likely responsible for the formation of carbon films. CVD is traditionally used to prepare carbon films and resulted in a decrease of strength. However, this new and inexpensive method allows for the transformation of the surface layer of carbides into carbon instead of depositing a film from a solution. The two major advantages of this technique is that the carbon films can be grown on top of different kind of SiC materials (powders, platelets, fibers and single-crystals) and its thickness can vary from 10–20 nm up to 1–2 μm, depending on the experimental conditions. In fact, the following parameters strongly influence the hydrothermal treatment of SiC:

− The temperature and time of treatment can affect the composition of the carbon film from amorphous to graphitic carbon,
− Above a certain temperature and reaction time, the yield of carbon reaches a maximum value,
− Above a certain temperature, the carbon film is oxidized,
− The influence of H_2_O:SiC is not well understood.

Indeed, thermodynamic calculations showed that the formation of a carbon deposit on the surface of SiC follows three regimes depending on the H_2_O:SiC molar ratio [128]:

1. *At low H_2_O:SiC molar ratios (1:10)*, both carbon and silica were deposited,
2. *At intermediate H_2_O:SiC molar ratios (2:1)*, both carbon and silica were produced, but silica is dissolved in the water as follows (70):

SiO_2_ + H_2_O = H_2_SiO_3_ = HSiO_3_^−^ + H^+^ = SIO_3_^2−^ + 2H^+^ (70)

A dissolution rate of approximately 0.8 μm was recorded for glassy silica in distilled water at 285 °C under 100 MPa pressure [129], meaning that the equilibrium of reactions (68) and (69) can be shifted to the right. Thus, a carbon-rich layer is created on top of the SiC materials.

3. *At higher H_2_O:SiC molar ratios (10:1)*, neither carbon nor silica was identified on the surface of SiC (for a nanoscale detection limit) as the carbon reacts with water to form CO/CO_2_ and silica dissolves in water.

Indeed, due to corrosion under supercritical conditions, water is able to oxidize the carbon coating according to the following reactions:

C + H_2_O → CO + H_2_ (water-gas reaction) (71)

2C + 2H_2_O → CO_2_ + CH_4_ (72)

C + 2H_2_ → CO_2_ + 2H_2_ (73)

3C + 2H_2_O → 2CO + CH_4_ (74)

Generally, for long corrosion times and intermediate H_2_O:SiC molar ratios, the carbon film at the surface is oxidized, and the oxidation is followed by silica deposition [130].

Gogotsi and Yoshimura investigated the behavior of SiC materials under dry and wet conditions. First, low-temperature oxidation (850 °C) was performed on SiC (Tyranno) fibers for up to 300 h [131]. The Tyranno fiber consisted of amorphous Si-Ti-C-O material with different oxygen contents (12 or 18 wt%), diameters (8.5 or 11 µm) and mechanical properties. This fiber contained silicon carbide crystals embedded in an amorphous matrix (Si-C-O-Ti). The oxidation started above 500 °C and led to a mass gain and an increase of the fiber diameter. Above 1200 °C, the mass gain increased sharply due to high diffusion rates of oxygen and carbon oxides through the silica layer.

**Figure 39:**
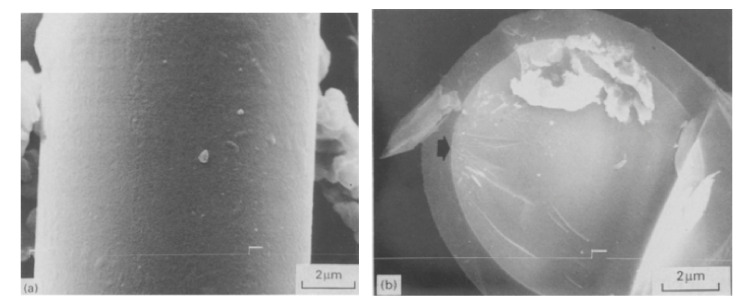
SEM micrographs of the surface (**a**) and fracture surface (**b**) of the S fibers oxidized for 300 h in air at 800 °C. The arrow shows the fracture origin. Reprinted with permission from Reference [131]. Copyright 1994 Springer Nature.

In Figure 39 the SEM micrographs of S fibers oxidized in air for 300 h at 800 °C are represented.

**Figure 40:**
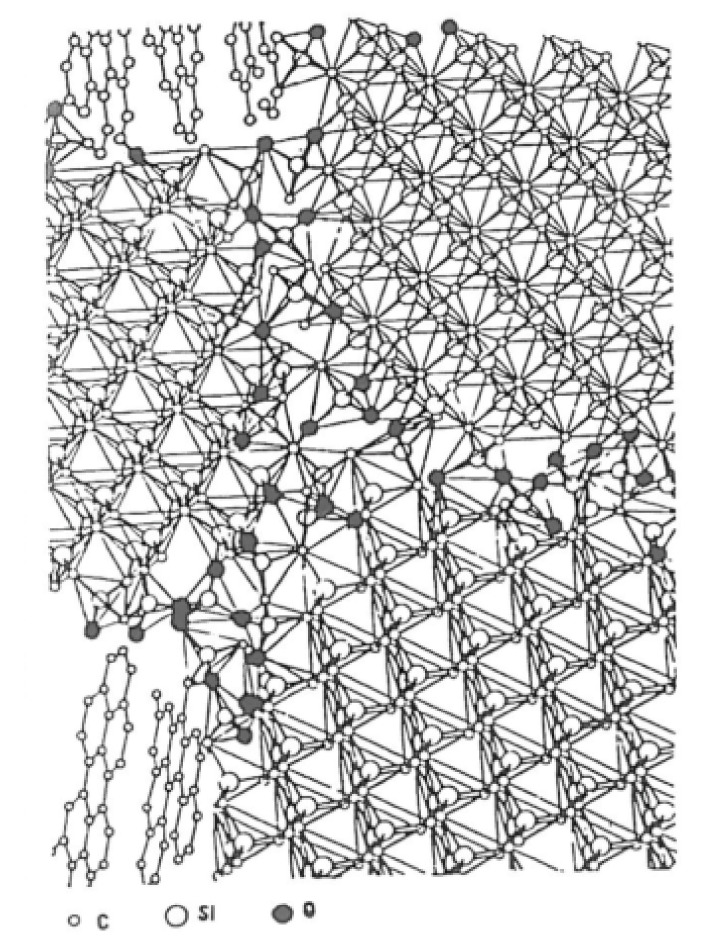
Texture model of the Nicalon NLM-202 fibers. Reprinted with permission from Reference [138]. Copyright 1999 Elsevier.

The thin, uniform (Figure 38a), glassy oxide layer formed (Figure 40b, in dark) was not dense and/or thick enough to limit the flaws on the surface. Moreover, the layer had low mechanical strength and showed cracking after mechanical tests (Figure 40b). As a result, the strength of the fibers decreased with increasing oxidation temperature/thickness of the oxide layer. This could be due to the decrease of the effective fiber diameter, which resulted from the oxide formation, and/or due to internal stress in the oxide layer.

As the oxide film on the surface was microscopically uniform, the oxidation is thought to be simultaneous for all constituents of the fiber [132]. Indeed, AES demonstrated the presence of a transition region at the SiC/SiO_2_ interphase, where silicon oxycarbide and free carbon could be produced, in accordance with other research [133].

Secondly, Gogotsi and Yoshimura observed the degradation of SiC (Tyranno) fibers in high-temperature and high-pressure water [134] for which amorphous or microcrystalline carbon constituted the carbon rich layer. The reaction leading to the formation of carbon (75):

SiC + 2H_2_O → SiO_2_ + C + 2H_2_ (75)

dominates over the reactions which produces silica (76), (77) and (78).

SiC + 2H_2_O → SiO_2_ + CH_4_ (76)

SiC + 4H_2_O → SiO_2_ + CO_2_ + 4H_2_ (77)

SiC + 3H_2_O → SiO_2_ + CO + 3H_2_ (78)

The interaction of SiC fiber with water was studied by Kraft et al. [135], between 400 and 700 °C, under 200 MPa water. However, the experiments were conducted at very low H_2_O: SiC molar ratio (1:10) and the formation of silica dominates in accordance with Yoshimura et al. [92]. However, at higher molar ratios, hydrosoluble silica is formed and the experiments agree well with the Hirayama’s model [92]. Finally, Gogotsi and Yoshimura demonstrated that oxidation and hydrothermal corrosion competition depends mostly on the temperature.

At 300 °C, the dissolution of the SiC_x_O_y_ and silica phases start, as AES depth profiles revealed no oxygen atoms at the surface of the fiber. The low consumption of SiC materials does not affect its strength properties [132].

Then, at 400 °C, poor low protective carbon film is produced by the following reaction (79):

SiC_x_O_y_ + nH_2_O → SiO_2_ + xC + nH_2_ (79)

Thus, the oxycarbidic phase of the Tyranno fibers starts to corrode, so the tensile strength and Young’s modulus decrease with the effective diameter of the fiber.

At 500 °C, the hydrothermal corrosion of crystalline SiC grains leads to the formation of thick carbon coatings (80):

SiC + 2H_2_O → SiO_2_ + C + 2H_2_ (80)

The layer demonstrates high protective and good adhesion properties. Above 800 °C, silica starts to crystallize into cristobalite and quartz, which have lower solubility in water. Therefore, the carbon film growth is limited, and hydrothermal treatment at high temperature and longer times engender the full consumption of the fiber.

Futatsuki et al. [136] studied the oxidation of SiC surface at low temperature and high-pressure water. They explored SCW (400 °C, 25 MPa) and SubCW (350 °C, 16.5 MPa) conditions to set up a high–efficiency method of oxidation.

The density of high-pressure and high-temperature water is in the range of 100–600 kg/m^3^, which is greater than the oxygen density under typical thermal oxidation conditions of 1200 °C, at atmospheric pressure, which is 0.003 kg/m^3^. The SC medium has a high oxygen solubility, and, due to its low viscosity and surface tension, its high diffusivity contributes to a strong oxidation potential.

They found out that the oxide thickness increases with the oxidation time, according to the following reactions:

SiC + 3/2 O_2_ → SiO_2_ + CO (81)

SiC + 2O_2_ → SiO_2_ + CO_2_ (82)

SiC + H_2_O → SiO_2_ + 3H_2_ + CO (83)

SiC + H_2_O → SiO_2_ + 4H_2_ + CO_2_ (84)

However, the oxidation of SiC was not possible without adding oxygen to water, so reactions (83) and (84) did not happen. When oxygen was added, they remarked that, for the NCW oxidation, the oxide film was very thin. Thus, the temperature might be too low to promote SiC oxidation. For SCW, oxidation occurs according to reactions (81) and (82).

Finally, the conclusions on this matter are as follows:

➔ SCW can facilitate the transfer of carbon by-products (CO and CO_2_) because of its high diffusivity. Therefore, the transfer of carbon by-products is one of the key factors promoting SiC oxidation.
➔ For NCW, the temperature is too low for the release of carbon by-products, so no SiC oxidation occurs.

However, NCW demonstrates high ionic constant which promotes hydrolysis reaction via Si atom etching. Recently, Yoko et al. [136] recycled silicon from silicon sludge by using SCW in a semi-batch reactor (400 °C, 25 MPa). It was noticed that silicon undergoes oxidation and/or degradation via Si dissolution into water. Indeed, between 260 and 380 °C, the amount of dissolved silicon increased with the temperature, whereas between 400 °C and 500 °C, it decreased. For temperatures higher than 1000 °C, the oxidation rate of silicon dominates. This behavior may be due to the low ion product of water between 400 and 500 °C under a pressure of 25 MPa.

According to Yoko et al. [137] and Futatsuki et al. [136], it seems how oxidation and hydrolysis compete depends not only on the temperature, but also on the ionic product. Indeed, NCW between 250 and 350 °C allows the etching of Si atoms, which dominates over oxidation.

### 5.3. Nanoporous Carbon Film Formation

As SiC materials are available in a continuous-fiber shape, they are promising to integrate into ceramic matrices for thermomechanical reinforcement properties. Therefore, it is necessary not only to improve the SiC corrosion resistance, but also to tune it in order for SiC surface to demonstrate new properties.

Two different fibers made of SiC are commercially available. The Nicalon fiber is obtained by polymerization of organosilane monomers followed by pyrolysis via the Yajima’s method [2]. At the end of the process, a microcomposite material is obtained which is made of a crystalline β-SiC phase (55 wt%), an intergranular oxycarbidic phase (40 wt%) and free carbon (5 wt%) [138] (Figure 40):

The other commercially available fiber is the Tyranno fiber which is made by pyrolysis of an organometallic polymer precursor synthetized from polycarbosilane and titanium tetrabutoxide [139]. At the end of the process, the continuous fiber consists of SiC crystals separated by an amorphous Si-Ti-C-O phase. Then, the Nicalon and Tyranno share the same texture model, which is described in Figure 40.

**Figure 41:**
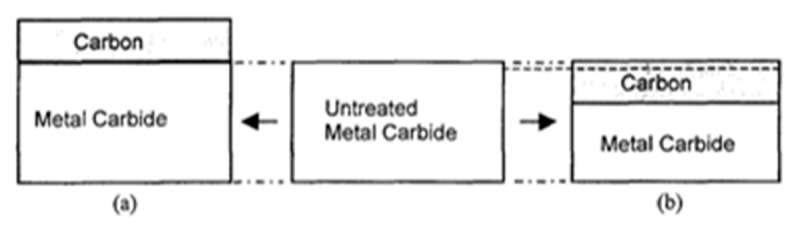
Carbon coating via vapor deposition (**a**) and selective leaching of carbides (**b**). Reprinted with permission from Reference [140]. Copyright 2000 Springer Nature.

To have good reinforcement properties, the fiber should demonstrate good compatibility with the matrix. For that, a carbon coating at the fiber/matrix interface is suitable in order for the composite to have a non-fragile behavior when submitted to stress. Gogotsi et al. have studied the hydrothermal treatment of carbon-based surfaces to form such a nanostructure carbon coating, as schematized below [140]. Under supercritical conditions, the selective etching of metals from carbides leads to the formation of a carbon-rich layer with tribological properties. This allows the fiber-reinforced materials to maintain high thermomechanical properties and therefore have the potential to be used in the aerospace field. The principle is represented in Figure 41.

Similar to the chemical or physical vapor deposition method, the carbon coating is grown on the surface, thus avoiding any adhesion issues.

**Figure 42:**
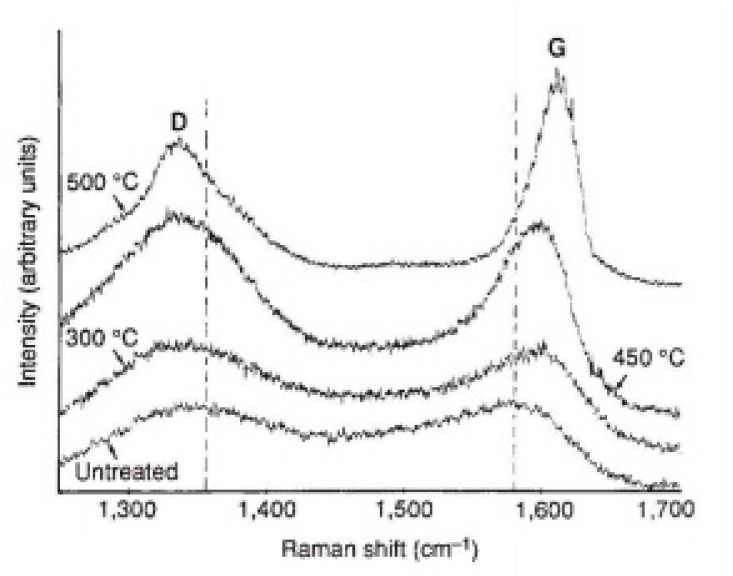
Raman spectra of the LoxM-garde Tyranno fibers before and after hydrothermal treatments at various temperatures. Ar laser radiation at a wavelength of 488 nm was used. Vertical dashed lines show the predicted positions of the nanocrystalline graphite line (G) at ~1580 cm^−1^ and its disorder-induced line (D) at ~1355 cm^−1^. The shift of the observed lines indicates the presence of bond-angle disorder. Reprinted with permission from Reference [126]. Copyright 1994 Springer Nature.

Gogotsi et al. showed that sp^3^-bonded carbon could be produced on the surface of SiC materials under hydrothermal conditions [141,142]. Various allotropes of carbon were obtained, including diamond, in the temperature range of 300–800 °C and at pressures below 500 MPa. Raman, infrared (IR), electron diffraction (EDS) and Auger Electron Spectroscopy (AES) were performed in addition to X-Ray Diffraction (XRD) to characterize treated and untreated materials. Depending on the shifting or the splitting of the G and D bands, their width and position, the Raman spectroscopy suggests the presence of disordered diamond and other non-graphitic carbon phases. The Raman spectra of SiC fibers before and after the hydrothermal treatment are given in Figure 42 [126]:

Graphite exhibits one Raman peak at 1580 cm^−1^ referred to as G mode, which is characteristic of the C=C stretching vibrations, whereas another peak appears at 1350 cm^−1^, which is referred to as D mode, when defects are present. This mode also appears for nanocrystalline graphite, and its intensity increases with the hydrothermal treatment temperature. The bands can broaden if strained bonds and defects are present in the materials. For the D band down-shift, Gogotsi at al. concluded that bond angle disorder came from strained sp^3^ carbon atoms. The up-shift of the G band towards the high-frequency edge, however, arose from small size crystallites, which are dominated by sp^2^-hydridized carbon rather than sp^3^ [143].

The IR spectroscopy assumes that H-bonded and free sp^3^-hybridized carbons were produced. The reflections at 2.06 Å was assigned to diamond on XRD patterns, whereas the narrow diamond Raman peak was missing, and the authors could not claim the presence of well-ordered cubic diamond. Indeed, the diamond sharp peak at 1332 cm^−1^ [144] is difficult to determine by conventional visible wavelength Raman spectroscopy when it is mixed with graphite. To have better sensitivity, UV excitation Raman spectroscopy could be used [145].

Finally, several key points could explain the formation of sp^3^-bonded carbons instead of simple amorphous or graphitic carbon network [142]:

(1) The presence of a good substrate: by acting as a template, the cubic structure of β-SiC could allow the diamond growth,
(2) The formation of hydrogen during hydrothermal treatment of SiC suggests that diamond was produced as hydrogen plays a role in the nucleation during diamond growth,
(3) Tetrahedral carbon in SiC is believed to be transformed into diamond and not into graphite for energetical reason,
(4) Preferential oxidation of sp^2^-bonded carbon by water seems to lead to the formation of carbon nuclei if the reaction (82) is replaced by the two following ones (85) and (86):

SiC + 2H_2_O → SiO_2_ + C + 2H_2_ (85)

SiC + 2H_2_O → SiO_2_ + C_(Si)_ + 4H^•^ (86)

**Figure 43:**
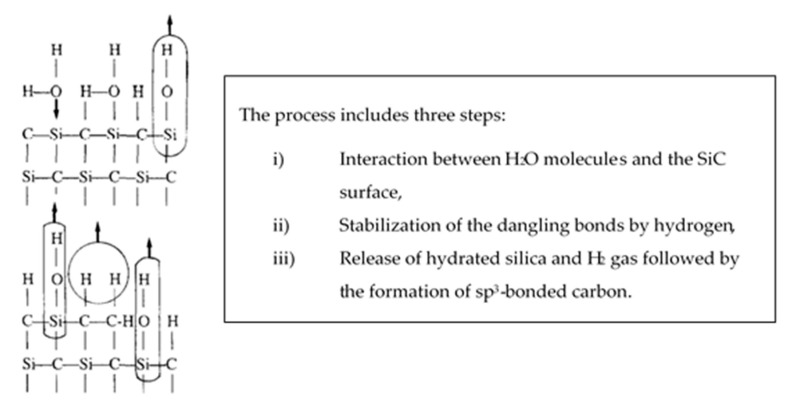
Two-dimensional sketch of the reaction mechanism for producing diamond. Reprinted with permission from Reference [142]. Copyright 1996 Elsevier.

C_(Si)_ + 4H^•^ → C_(diamond)_ + 2H_2_ (87)

where C_(Si)_ is a carbon atom that has at least one bond with a Si atom which could act as a nucleus for diamond growth following the mechanism below, on Figure 43.

By comparison with the model of the SiC etching via chlorine gas, suggested by Peng et al. [146], it seems that both mechanisms are similar. However, under high pressure conditions, it seems that the carbon-rich layer has a higher chance to evolve into sp^3^-hybridized structure, which is diamond [142] whereas at ambient pressure, it gives rise to few layers of graphene.

Moreover, carbon formation could be a consequence of the three following processes:

○ From the free carbon (USB) located around the SiC crystals, in Nicalon and Tyranno fibers,
○ From a C-H-O-(Si) fluid which is created under hydrothermal conditions [129],
○ The growth of the diamond particles could be due to the reaction of carbonaceous by-products (88) in a H_2_O-dominated fluid [147]:

CO_2_ + CH_4_ → 2C + 2H_2_O (88)

As CH_4_ and CO_2_ gas are not stable at high temperature, the reaction is thermodynamically possible [148].

To conclude, multiple parameters, such as the composition of the hydrothermal media and the temperature and pressure conditions, allow for the control of the nature of the carbon layer, along with its properties.

Likewise, it was shown that the molar ratio and time of treatment is an important factor during the hydrothermal treatment as hydrolysis or oxidation processes can be favored for thermodynamic and/or kinetic reasons. As a result, the SCW treatment is efficient for producing either carbon films or silica films, and, sometimes, neither film.

### 5.4. The Use of Silicon Carbide in the Nuclear Field

Recently, silicon carbide materials have attracted attention in the nuclear field. Since the Fukushima Daishi incident in Japan in 2011, replacement of Zircaloy cladding in Pressurized Water Reactors (PWR) has been an important issue. SiC materials constitute an alternative due to their excellent thermomechanical properties, accident tolerance and resistance to irradiation [149]. Moreover, the development of TRISO particles, as alternative fuel material is of great interest [150]. Therefore, information and comprehension about the behavior of SiC materials in high temperature and high pressure water is necessary. More precisely, factors influencing the material’s resistance to high pressure water oxidation have been investigated, as well as solutions to improve this resistance.

Terrani et al. [151] studied the corrosion of CVD-SIC and SiC composites materials in conditions similar to Light Water Reactor (LWR) operating conditions (290 ≤ T ≤ 330 °C). From a chemical-mechanism point of view, they did not observe any silica on the surface, suggesting that it was dissolved by the water media. In accordance with Hirayama’s model, the recession of SiC was limited by surface reaction, i.e., silica formation on the surface of SiC.

SiC + O_2_ → SiO_2_ + CO_2_ (89)

Moreover, oxygen activity in the system is believed to play a major role, as it is the chemical species responsible for SiC oxidation. Indeed, increasing oxygen activity (from 2.0 × 10^−36^ to 1.7 × 10^−6^) leads to an important increase of reaction rate (from 7.3 × 10^−9^ to 4.43 × 10^−7^). It is important to note that oxygen activity is directly related to irradiation of the media, so irradiation is directly responsible for important corrosion of the silicon carbide phase [152].

On the contrary, dissolved hydrogen is believed to have the opposite effect [153], as it reduces the chemical affinity of the chemical reaction forming SiO_2_. More precisely, H_2_ is believed to act as a scavenger of O_2_, which supposedly decreases oxygen activity in the media.

Doyle et al. [154] established a model to determine SiC recession rate according to roughness, amount of oxygen dissolved and temperature:

(90) $$\begin{array}{cc} \frac{0.1458}{1+SA}T & (1.09(1-10^{-3}T)\left[O_{2}\right]e^{\frac{-1.275*10^{4}}{T}\;}+7.91*\\ & 10^{-6}e^{-\frac{7.39*10^{3}}{T}} \end{array}$$

However, this model does not take into account a certain number of parameters or mechanisms, such as grain fallout or morphology of the sample (fibers, for example).

Shin et al. [155] gave another important parameter regarding the recession of SiC, which is electrical conductivity of the sample. Indeed, higher electrical conductivity of the sample leads to higher exchange current density and promotes electrochemical reactions. Because the formation of silica (or silicon hydroxide) is electrochemical in nature, a higher electrical conductivity of the sample induces an increase in the recession rate.

The behavior of SiC materials also obviously depends on their microstructure and their method of elaboration.

Manufacturing of dense silicon carbide monoliths often requires the use of sintering additives that can have a strong influence on the corrosion behavior of the material.

**Figure 44:**
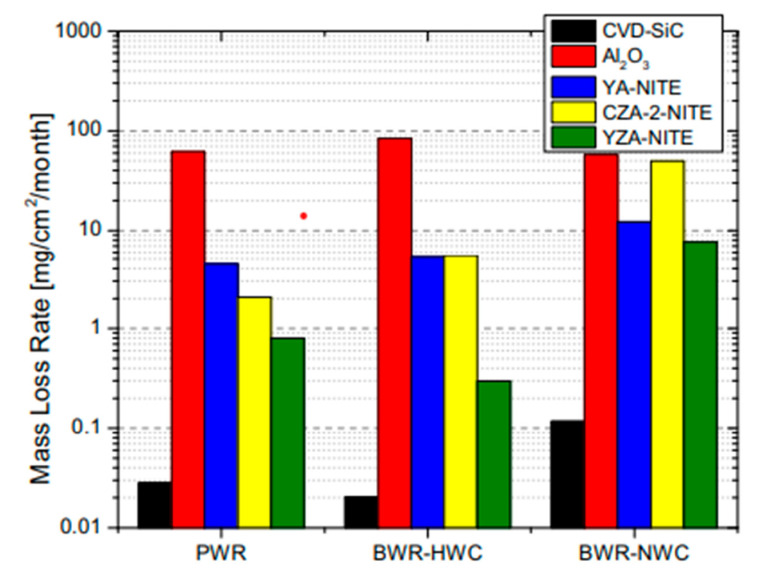
Evolution of mass loss for different SiC materials with different additives. Reprinted with permission from Reference [156]. Copyright 2017 Elsevier.

Parish et al. [156] studied the influence of different sintering additives for the NITE process (liquid phase sintering using nanopowder of SiC) (Figure 44).

Results of the study show that sintering additives greatly decrease the SiC material’s resistance toward oxidation. Additives used in this study include alumina, ceria and zirconia. Alumina, which is the most used sintering additive in NITE process, display such high recession rate that the material disappeared after two days of exposure to reactive media. In fact, the behavior of the sintered material depends on the affinity of the additive with water. Alumina is rapidly dissolved by water, thus leaving the grain boundaries of the material open, which is catastrophic for corrosion resistance. On the contrary, yttria(Y_2_O_3_) used with alumina [157] forms a eutectic compound (Y_3_Al_5_O_12_) which acts as a protective barrier for corrosion. Similarly, adding chromium leads to the formation of chromium oxide, another protective oxide [158]. However, a precise balance between sintering additives and protective oxides needs to be considered to make the sintering possible while maintaining an acceptable corrosion behavior.

More precisely, Suyama et al. [159] precisely described the hydrothermal corrosion of the constituents of a SiC_f_/SiC composite.

**Figure 45:**
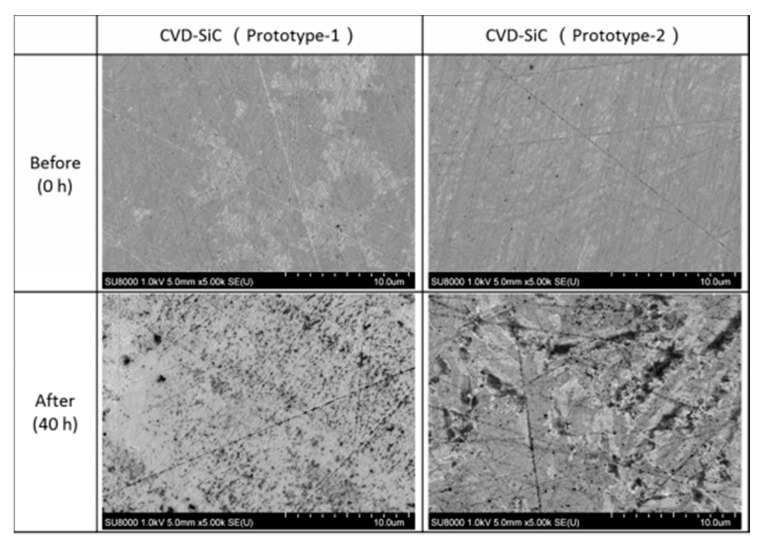
SEM images of CVD-SiC before and after corrosion testing. Reprinted with permission from Reference [159]. Copyright 2019 MDPI AG/Creative Commons Attribution License 4.0.

As expected, silicon carbide fibers, whatever purity or crystallinity, were affected by hydrothermal corrosion. No hypothesis was made about the mechanism, but it is highly probable that silica was formed and dissolved by the media. Likewise, the SiC matrix did undergo corrosion, which was mainly affected by the surface state of the sample (Figure 45).

To conclude, recent studies on hydrothermal corrosion of silicon carbide focused on the use of SiC materials in nuclear environments. The mechanism of hydrothermal corrosion has been widely studied in the literature, and very accurate models can be used. However, the effect of the chemical structure of the candidate materials needs to be elucidated in order to optimize the materials elaboration and maximize the corrosion resistance.

### 5.5. Conclusions

Hydrothermal corrosion via hydrolysis leads to the formation of a carbon-rich layer by Si etching whereas the hydrothermal oxidation leads to the formation of silica. These two processes compete depending both on the temperature and the H_2_O:SiC molar ratios, as follows:

As shown, for high H_2_O:SiC molar ratios, silicon carbide is hydrolyzed by water without carbon formation. However, by increasing the temperature, the hydrothermal oxidation is favored, and silica and carbon are formed. For low molar ratios, no hydrolysis is observed, whereas above 500 °C, the oxidation of SiC starts. Thus, carbon and silica are recovered on the SiC surface, as silica does not dissolve. In this case, increasing the temperature does not affect the carbon film, whereas for higher molar ratios, carbon is oxidized by water above 600 °C.

Carbon coating can find applications in many fields as it improves ceramics’ sinterability and allows for control over electrical conductivity. Indeed, graphitic carbon can decrease friction coefficients for lubricating applications, whereas diamond coating demonstrates high hardness for abrasive applications.

Finally, the improvement of the fiber/matrix compatibility is essential for ceramic composites to have a ductile behavior when submitted to stress. For maximizing the compatibility, the coating should be tailored in term of composition, structure, porosity and thickness. [160].

Understanding the numerous chemical phenomena involved in hydrothermal corrosion of SiC has proven to be useful in the design of materials for harsh environments, such as a nuclear reactor. Most recent studies therefore focus on the behavior of SiC or SiC composite materials in hydrothermal conditions typical for the new generation of reactors, studying the effect of experimental conditions and microstructure of the materials (summarized in Table 8).

## 6. Conclusions

First, Deal and Grove established a kinetic model for the silicon oxidation under wet and dry atmosphere [11] and parabolic and linear regimes were defined for expressing the growth of silica. The parabolic regime expresses a diffusion-controlled mechanism whereas the linear one expresses a surface-controlled mechanism. As this model fits the oxidation behavior of silicon and silicon carbide materials, it was widely used for expressing the silica growth on SiC materials under dry and wet conditions.

It should be mentioned that recently, Hijitaka et al. [18] developed and proved the effectiveness of the Si an C emission model to explain the SiC oxidation for the whole thickness of oxide.

For both processes, dry and wet (air) oxidation, the kinetics show time-dependence. Indeed, for short oxidation time, a thin amorphous oxide film is created, and the growth of silica is linear, whereas at longer time, a thick film is created for which the kinetics follow the parabolic regime. The parabolic regime shows a temperature-dependence, as the amorphous silica crystallizes with the temperature. Indeed, tridymite, cristobalite and quartz phases show lower permeability to oxidant species and make the diffusion process slower. This, in turn, slows the oxidation rates.

From a microstructural point of view, differences between pure dense SiC and SiC within other microstructures should be emphasized. As SiC oxidation is occurring at grained boundaries first, different behaviors can be observed, depending on the microstructure of the material.

When adding water to the system of C-O-Si, oxidation and subsequent degradation of SiC can be drastically enhanced, even with small amounts of H_2_O. Indeed, water has the capacity to loosen the oxide network due to its high reactivity with silanol bonds, which allows a faster solution and diffusion of the molecule through the scale. In fact, the primary oxidant during wet air oxidation is water, but oxygen molecules diffuse as well. Therefore, a kind of synergy is developed as water facilitates the transport of oxidant species through the scale and oxygen demonstrates high reactivity with the Si-C bonds. That is why a new oxidation regime is defined, the paralinear regime, for which porous and non-protective scales are obtained due to high oxide volatilization.

When high temperature and high-pressure water interacts with SiC materials, chemical corrosion occurs. Three models were proposed, and the microstructure study of the corroded SiC surface shows agreement with the Allongue’s model [93]. Indeed, he proposed that successive hydrolysis of the Si-Si and Si-H bonds was involved in the corrosion process of silicon crystals. As no silica scale was found at the surface, Hirayama proposed the formation of hydrosoluble silica, which is pH-dependent [92]. From that aspect, presence of alkaline ions (Na for example) can also dramatically increase corrosion by promoting dissolution of silica scale.

The oxidation behavior of SiC seems complex, as several reactions compete along with different corrosion processes, as is suggested by the tribochemical corrosion model of Presser et al. [106].

However, the corrosion of SiC can be controlled using SCW due to its high tunability. By varying the temperature, SCW demonstrates high solvent power for organic, salts or gases and from liquid-like to gas-like properties. Gogotsi and Yoshimura [125] created a carbon coating on top of SiC materials by optimizing the properties of high temperature and high-pressure water. Its high reactivity allows the etching of Si atoms which leads to the carbon enrichment of the SiC surface; however, high temperature leads to the oxidation of carbon. As SCW shows high diffusivity, the thickness of the layer evolves linearly with time. Finally, the H_2_O:SiC molar ratio has great influence, as it favors either hydrolysis or oxidation reactions. Indeed, low ratios lead to the oxidation of SiC and silica and carbon are created. However, high ratios lead to the oxidation of SiC with creation of carbon. The hydrothermal corrosion of SiC has therefore been the subject of an extensive study in the literature, and mechanisms have been elucidated. That is why recent studies on the supercritical oxidation of SiC are focused on the influence of the structure of the material (composition, architecture) and on its corrosion by high pressure, high temperature water.

## Figures and Tables

**Table 1:** Activation energies for linear oxidation of SiC in dry atmosphere.

Types of SiC	T (°C)	Kinetics	Activation Energy for Linear Oxidation (kJ/mol)
Powder SiC (green) short heating and cooling cycles	1100	Linear law	No data [25]
Si slices (111)	900–1200	Linear parabolic	193 [11]
Single crystals SiC (thin oxide face)	970–1245	Linear parabolic	355 [12]
RF-Sputtered thin α-SiC films (C-face)	950–1100	Linear law	155–200 [26]
Single crystal Si	800–1100	Linear parabolic	155 [27]
Single crystal SiC (fast oxidation face)	800–1100	Linear parabolic	159 [27]
Single crystal SiC (slow oxidation face)	800–1100	Linear parabolic	330 [27]
CVD-SiC thick films (fast oxidation face)	800–1100	Linear parabolic	170 [27]
CVD-SiC thick films (slow oxidation face)	800–1100	Linear parabolic	334 [27]

**Table 2:** Activation energies for parabolic oxidation of SiC in dry atmosphere.

Single crystals SiC (thick oxide face)	970–1245	Linear parabolic	196 [12]
HfB2 + 20 v/o SiC composite	1350–1550	Parabolic law	452 [6]
Hot-pressed SiC	1200–1400	Parabolic law	481 [28]
Hot-pressed SiC	1200–1500	Parabolic law	134–389 [29]
Sintered α-SiC	1200–1500	Parabolic law	155–498 [29]
Single-crystals Si	1200–1400	Linear parabolic	120 [30]
Single-crystal SiC (green) (fast-grow face)	1200–1400	Linear parabolic	121–297 [30]
Single-crystal SiC (green) (slow-grow face)	1200–1400	Linear parabolic	339 [30]
Controlled nucleation thermally deposited SiC	1200–1400	Linear parabolic	142–293 [30]
Sintered α-SiC	1200–1400	Linear parabolic	217–289 [30]
Hot-pressed SiC	1200–1400	Linear parabolic	221 [30]
CVD-SiC	1550–1675	Linear parabolic	345 (amorphous silica) and 387 (cristobalite) [31]
Single crystal SiC (green) (C face)	1200–1350	Parabolic law	120 [9]
Single crystal SiC (green) (C face)	1350–1500	Parabolic law	260 [9]
Single crystal SiC (green) (Si face)	1350–1500	Parabolic law	223–298 [9]
CVD-SiC	1200–1400	Linear parabolic	142 [32]
Single crystal Si	800–1100	Linear parabolic	96 [27]
Single crystal SiC (fast oxidation face)	800–1100	Linear parabolic	99 [27]
Single crystal SiC (slow oxidation face)	800–1100	Linear parabolic	292 [27]
CVD-SiC thick films (fast oxidation face)	800–1100	Linear parabolic	94 [27]
CVD-SiC thick films (slow oxidation face)	800–1100	Linear parabolic	285 [27]
CVD-SiC	1200–1500	Linear parabolic	118 [33]
CVD-SiC	1397–1737	Linear parabolic	210 [34]
**Types of SiC**	**T (°C)**	**Kinetics**	**Activation energy for parabolic oxidation (kJ/mol)**
Powder SiC (black)	1000–1200	Parabolic law	209 [35]
Powder SiC (green) Oxidation time <30 min	1000–1200	Parabolic law	117 [35]
Powder SiC (green) Oxidation time >60 min	1000–1200	Parabolic law	263 [35]
Powder SiC (green) short time oxidation	1100–1300	Parabolic law	209 [25]
Single-crystals SiC (green)	1200–1500	Parabolic law	276 [36]
High purity SiC	900–1200	Parabolic law	85 (amorphous silica) and 65 (cristobalite) [37]
High purity SiC	1380–1556	Parabolic law	190 [38]
Si slices (111)	900–1200	Linear parabolic	119 [11]
Si slices (111)	1000–1200	Parabolic law	125 [39]
Powder SiC	1200–1500	Parabolic law	632 [40]
Polycrystalline CVD SiC	1477–1627	Linear parabolic	1130 [41]
Self-bonded SiC (50/50 α/β)	1000–1300	Parabolic law	No data [42]

**Table 3:** Activation energies for linear oxidation of SiC in wet atmosphere.

Types of SiC	Oxidant Species	T (°C)	Kinetics	Activation Energy for Linear Oxidation (kJ/mol)
Si slices (111)	Wet O_2_	900–1200	Linear parabolic	190 [11]
Powder SiC	Wet air	1200–1400	Linear law	146 [57]
Powder SiC	Wet O_2_, Ar, N_2_	1500	Linear law	No data [70]
Single-crystal α-SiC (Si + C faces)	84% vol H_2_O	850–1050	Linear parabolic	109 [51]
Single-crystal α-SiC (C face)	84% vol H_2_O	850–1050	Linear parabolic	200 [51]
Single-crystal β-SiC (C face)	98 °C water + O_2_	1000–1200	Linear parabolic	251 [69]
Single-crystal β-SiC (C face)	98 °C water + Ar	1000–1200	Linear parabolic	280 [69]
CVD-SiC	84% vol H_2_O	1000–1250	Linear parabolic	309 [71]
RF-Sputtered thin α-SiC films	84% vol H_2_O	950–1100	Linear law	205–218 [25]
CVD-SiC	10% H_2_O in O_2_	1550–1650	Linear parabolic	428 [72]
Powder-SiC	50%H_2_O/50%O_2_	1200–1400	Parabolic law	No data [61]
Sintered α-SiC	12.3% H_2_O, 2.1% O_2_, 11.0% CO_2_, 71.8% N_2_ 50% H_2_O/50% O_2_	1316	Paralinear	No data [63]
CVD-SiC		1100–1400	Paralinear	No data [65]
Sintered α-SiC	50%H_2_O/50%O_2_	1100–1400	Paralinear	No data [65]
Fused quartz	50%H_2_O/50%O_2_	1100–1400	Paralinear	No data [65]

**Table 4:** Activation energies for parabolic oxidation of SiC in wet atmosphere.

Types of SiC	Oxidants	T (°C)	Kinetics	Activation Energy for Parabolic Oxidation (kJ/mol)
High purity SiC	H_2_O/Ar	1218–1514	Parabolic law	102 [46]
Si slices (111)	Wet O_2_	900–1200	Linear parabolic	68 [11]
Si slices (111)	90 °C water + O2	1000–1200	Parabolic law	85 [40]
Si slices (111)	Steam	1000–1200	Parabolic law	102 [40]
SiC (50/50 of α/β)	Wet O_2_	1000–1300	Parabolic law	No data [43]
Hot pressed SiC	3% H_2_O in O_2_	1200–1400	Parabolic law	527 [58]
Single crystals Si (100)	H_2_O/O_2_ (1 to 2000 ppm)	780–980	Linear parabolic	No data [62]
Single crystals Si (100)	H_2_O/N_2_ (1 to 2000 ppm)	780–980	Linear parabolic	No data [62]
Single-crystal β-SiC (C face)	98 °C water + O_2_	1000–1200	Linear parabolic	531 [69]
Single-crystal β-SiC (C face)	98 °C water + Ar	1000–1200	Linear parabolic	656 [69]
CVD-SiC	84% vol H_2_O	1000–1250	Linear parabolic	209 [71]
Pressureless-sintered α-SiC	H_2_O in Air (10 to 40% vol)	1300	Parabolic law	No data [73]
CVD-SiC	10% H_2_O in O_2_	1550–1650	Linear parabolic	397 [74]
CVD-SiC in fused quartz tubes	10% H_2_O in O_2_	1200–1400	Linear parabolic	41 [32]
CVD-SiC in high purity Al_2_O_3_ tubes	10% H_2_O in O_2_	1200–1400	Linear parabolic	249 [32]
CVD-SiC	H_2_O/O_2_ (10 to 90% vol)	1200–1400	Parabolic	28-156 [66]
CVD-SiC	H_2_O/Ar	1200–1400	Parabolic	No data [66]
Si, Sintered α-SiC, CVD-SiC	Air + 15% vol H_2_O	1200	Paralinear (adapted from the model of Haycock)	No data [67]

**Table 5:** Water partial pressure dependence for several water vapor defect species using standard Kroger–Vink notation (from Reference [64]).

Water Vapor Defect Species	Defect Formation Reaction	Mass Action Expression	Electro-Neutrality Expression	Water Vapor Partial Pressure Dependence	Power Low Exponent for Water
$H_{2}O_{i}^{x}$	$H_{2}O\left(g\right)=H_{2}O_{i}^{x}$	$K_{1}=\left[H_{2}O_{i}^{x}\right]/P_{H2O}$	none	$\left[H_{2}O_{i}^{x}\right]\propto P_{H2O}^{1}$	1
no	$H_{2}O\left(g\right)=H_{2}+\frac{1}{2}O_{2}$	$K_{2}={\left[O_{2}\right]}^{1/2}P_{H2}/P_{H2O}$	none	O2∝PH2O2PH22	2
$HO_{i}^{\prime }$	$H_{2}O\left(g\right)=HO_{i}^{\prime }+H_{i}^{˙}$	$K_{3}=\left[HO_{i}^{\prime }\right]\left[H_{i}^{˙}\right]/P_{H2O}$	$\left[HO_{i}^{\prime }\right]=\left[H_{i}^{˙}\right]$	$\left[HO_{i}^{\prime }\right]\propto P_{H2O}^{1/2}$	1/2
$O_{i}^{”}$	$H_{2}O\left(g\right)=2H_{i}^{˙}+O_{i}^{”}$	$K_{4}=[O_{i}^{”}]{\left[H_{i}^{˙}\right]}^{2}/P_{H2O}$	$\left[O_{i}^{”}\right]=\left[H_{i}^{˙}\right]$	$\left[O_{i}^{”}\right]\propto P_{H2O}^{1/3}$	1/3
$O_{i}^{”}$	$H_{2}O\left(g\right)=2h_{i}^{˙}+O_{i}^{”}+H_{2}$	$K_{5}={\left[h_{i}^{˙}\right]}^{2}[O_{i}^{”}]P_{H2}/P_{H2O}$	$\left[O_{i}^{”}\right]=\left[h_{i}^{˙}\right]$	Oi”∝PH2O1/3PH21/3	1/3

**Table 6:** Activation energy for water and oxygen molecules through silica and silicon-containing bond energies.

Material/Silicon Bonding	Activation Energy in the Parabolic Regime Which Is Limited by Diffusion of Oxidant Species through Silica				Linear Regime Limited by the Interface Reaction
	Conditions (°C)	Oxygen Permeation (kJ/mol)	Conditions (°C)	Water Permeation (kJ/mol)	Breaking Energy (kJ/mol)
Fused silica	950–1100	113 [44]	300–1100	70 [81]	
Cristobalite	1000–1400 on SiC substrate	430 [87]		/	
Tridymite	1070–1280	195 [88]		/	
β-Quartz	870–1180	195 [88]	600–800 + 100 MPA H_2_O	142 (//to c) [89]	
Si-Si					177 [73]
Si-C					290 [73]
Si-O or Si-OH					377 [81]

**Table 7:** Physicochemical properties of water as a function of temperature and pressure. Reprinted with permission from Reference [111]. Copyright 1999 Wiley.

	“Normal Water”	“Subcritical Water”	“Supercritical Water”		Superheated Steam
T [°C]	25	250	400	400	400
P[MPa]	0.1	5	25	50	0.1
ρ[g·cm^−3^]	0.997	0.80	0.17	0.58	0.0003
ε	78.5	27.1	5.9	10.5	1
pK_w_	14.0	11.2	19.4	11.9	/
C_p_[kJ.kg^−1^·K^−1^] η[mPa.s]	4.22 0.89	4.86 0.11	13 0.03	6.8 0.07	2.1 0.02
λ[mW·m^−1^·K^−1^]	608	620	160	438	55

**Table 8:** Competition between the hydrothermal oxidation and hydrolysis of crystalline silicon carbide, oxycarbidic and silica phases by water depending on the temperature and the H_2_O: SiC molar ratios. Extracted from References [92,93,126].

P = 10–100 MPa	Low (H_2_O:SiC) Molar Ratios (1: 10)	Intermediates (H_2_O:SiC) Molar Ratios (2: 1)	High (H_2_O:SiC) Molar Ratios (10: 1)
Observations	Deposition of carbon and silica, according to Yoshimura	Formation of a carbon layer and dissolution of silica, according to Gogotsi	Oxidation of carbon and dissolution of silica, according to Hirayama
300 °C	No reactions	SiC_x_O_y_ + nH_2_O → SiO_2_ +xC + nH_2_ SiO_2_ + H_2_O → SiO_3_^2−^ + 2 H^+^	SiC + 4 H_2_O → Si(OH)_4_ + CH_4_ SiC_x_O_y_ + 4 H_2_O → Si(OH)_4_ + xCH_4_
400 °C			
500 °C	SiC + 2 H_2_O → SiO_2_ + C + 2 H_2_ SiC + 2 H_2_O → SiO_2_ + CH_4_ O_2_ + CH_4_ → CO_2_ + 2 H_2_O	SiC + 2 H_2_O → SiO_2_ + C + 2 H_2_ SiC_x_O_y_ + nH_2_O → SiO_2_ +xC + nH_2_ SiO_2_ + H_2_O → SiO_3_^2−^ + 2 H^+^	SiC + 2 H_2_O → SiO_2_ + C + 2 H_2_ SiC_x_O_y_ + nH_2_O → SiO_2_ +xC + nH_2_ SiO_2_ + H_2_O → SiO_3_^2−^ + 2 H^+^
600 °C	SiC + 2 H_2_O → SiO_2_ + C + 2 H_2_ SiC + 3 H_2_O → SiO_2_ + CO + 3 H_2_ SiC + 4 H_2_O → SiO_2_ + CO_2_ + 4 H_2_	C + H_2_O → CO + H_2_ 2C + 2H_2_O → CO_2_ + CH_4_ 3C + 2H_2_O → 2CO + CH_4_	C + H_2_O → CO + H_2_ 2C + 2H_2_O → CO_2_ + CH_4_ 3C + 2H_2_O → 2CO + CH_4_

## Data Availability

Not applicable to this work.
